# Comparison of four healthy lifestyle scores for predicting cardiovascular events in a national cohort study

**DOI:** 10.1038/s41598-021-01213-6

**Published:** 2021-11-12

**Authors:** Ming-Chieh Tsai, Tzu-Lin Yeh, Hsin-Yin Hsu, Le-Yin Hsu, Chun-Chuan Lee, Po-Jung Tseng, Kuo-Liong Chien

**Affiliations:** 1 Institute of Epidemiology and Preventive Medicine, College of Public Health, National Taiwan University, Room 517, No.17, Xu-Zhou Rd., Taipei, 10055 Taiwan; 2 Division of Endocrinology and Metabolism, Department of Internal Medicine, Taipei Mackay Memorial Hospital, No. 92, Section 2, Zhongshan North Road, Taipei City, 10449 Taiwan; 3 Department of Medicine, Mackay Medical Collage, No. 46, Sec. 3, Zhongzheng Rd., Sanzhi Dist., New Taipei City, 25245 Taiwan; 4 Department of Family Medicine, Hsinchu MacKay Memorial Hospital, No. 690, Section 2, Guangfu Road, East District, Hsinchu City, 30071 Taiwan; 5 Department of Family Medicine, Taipei MacKay Memorial Hospital, No. 92, Section 2, Zhongshan North Road, Taipei City, 10449 Taiwan; 6 Division of Cardiovascular Surgery, Department of Surgery, Hsin Chu Armed Force Hospital, Hsinchu, Taiwan; 7 Department of Internal Medicine, National Taiwan University Hospital, No. 7, Zhongshan S. Rd., Zhongzheng Dist., Taipei City, 10002 Taiwan

**Keywords:** Risk factors, Cardiovascular diseases

## Abstract

The protective effect of different healthy lifestyle scores for the risk of cardiovascular disease (CVD) was reported, although the comparisons of performance were lacking. We compared the performance measures of CVDs from different healthy lifestyle scores among Taiwanese adults. We conducted a nationwide prospective cohort study of 6042 participants (median age 43 years, 50.2% women) in Taiwan’s Hypertensive, Hyperglycemia and Hyperlipidemia Survey, of whom 2002 were free of CVD at baseline. The simple and weighted the Mediterranean diet related healthy lifestyle (MHL) scores were defined as a combination of normal body mass index, Mediterranean diet, adequate physical activity, non-smokers, regular healthy drinking, and each dichotomous lifestyle factor. The World Cancer Research Fund and American Institute for Cancer Research cancer prevention recommended lifestyle and Life's Simple 7 following the guideline definition. The incidence of CVD among the four healthy lifestyle scores, each divided into four subgroups, was estimated. During a median 14.3 years follow-up period, 520 cases developed CVD. In the multivariate-adjusted Cox proportional hazard models, adherence to the highest category compared with the lowest one was associated with a lower incidence of CVD events, based on the simple (hazard ratio [HR] 0.43, 95% confidence interval [CI] 0.2–0.94) and weighted MHL scores (HR 0.44, 95% CI 0.28–0.68). Additionally, age played a role as a significant effect modifier for the protective effect of the healthy lifestyle scores for CVD risk. Specifically, the performance measures by integrated discriminative improvement showed a significant increase after adding the simple MHL score (integrated discriminative improvement: 0.51, 95% CI 0.16–0.86, *P* = 0.002) and weighted MHL score (integrated discriminative improvement: 0.38, 95% CI 0.01–0.74, *P* = 0.021). We demonstrated that the healthy lifestyle scores with an inverse association with CVD and reduced CVD risk were more likely for young adults than for old adults. Further studies to study the mechanism of the role of lifestyle on CVD prevention are warranted.

## Introduction

Cardiovascular diseases (CVDs) are the leading noncommunicable diseases and were associated with an estimated 17.9 million deaths (31% of the global annual mortality) in 2016^1,2^; moreover, 85% of the deaths were attributed to coronary heart disease-related myocardial infarctions and stroke (7.4 and 6.7 million, respectively). Individually, several modifiable lifestyle factors have been associated with reducing the odds of CVD. For example, Mediterranean diet had a lower risk of CVD within the range of 0.53–0.75 reported in previous studies^3–5^. Further research has explored a combination of multiple healthy lifestyle behaviors, rather than a single behavior, that facilitates processes beneficial to the prevention of CVD; a decrease in the overall incidence of CVD was found (range 0.22–0.45)^6–28^. In 2007, the World Cancer Research Fund/American Institute for Cancer Research (WCRF/AICR) reported lifestyle recommendations^29^ to potentially reduce cancer risk in adults based on a comprehensive meta-analysis that included more than 500 investigations. The American Heart Association defined “Life's Simple 7”—an evidence-supported effective construct of ideal cardiovascular health with four favorable health behaviors and three health clinical factors, including serum total cholesterol, blood pressure, and no diabetes, to improve the cardiovascular health in the community^30–37^.

Consensus on the combined lifestyle factors on primordial CVD prevention are still arguable. The above various lifestyle scores confuse health professionals, especially in non-Caucasian populations, such as with ethnic Chinese. First, we do not know the consistency and agreement among the above healthy lifestyle scores, especially applied in the population attribution fraction on the CVD incidence. Second, the combined lifestyle scores in previous studies were the sum of the non-weighted healthy behaviors based on the assumption that all lifestyle factors have the same magnitude of effect and that they potentially might lead to a misclassification bias. The impact of the weighted healthy lifestyle score on the CVD incidence has important clinical implications, although there is lacking evidence on this aspect. Moreover, the healthy lifestyle score derived from the recommendation of WCRF/AICR has been shown to favorably influence cancer risk, although there is little evidence on the CVD risk prediction. Additionally, Life's Simple 7 has an inverse association with the CVD risk, although whether the protective benefits are more from the clinical risk or from lifestyle factors is controversial. Finally, age as a potential effect modifier on the association between the healthy lifestyle score and CVD risk has been studied in secondary data^38^, although there has been no validation in research from primary analysis on whether targeting younger adults for the primordial prevention of CVD would be more feasible compared with older adults.

Accordingly, we sought to assess whether the healthy lifestyle scores, as captured by a simple and weighted combination of nonobese body mass index (BMI), healthy dietary quality, physical activity, non-smoking, and adequate drinking, are associated with CVD risk in a national representative cohort of Taiwanese adults, especially in young ones with low short-term risk. Further, the performance ability among the score comprised alternative Mediterranean diet and other lifestyle factors, i.e., the Mediterranean diet related healthy lifestyle (MHL) score (non-weighted and weighted) from the WCRF/AICR recommendation and Life's Simple 7 for predicting CVD risk, were compared.

## Methods

### Participants

The 2002 Taiwan Survey of Hypertensive, Hyperglycemia, Hyperlipidemia Survey (2002 TwSHHH) was a national representative prospective cohort established in 2002. Briefly, participants of the TwSHHH cohort were included on the basis of multistage, stratified, random sampling from the National Health Interview Survey, which recruited 7578 individuals; detailed information was obtained from a face-to face questionnaire, anthropometric measurements, and blood sample analyses, which has been described in previous articles^39,40^. Each participant’s baseline data collected from March 11, 2002 to August 10, 2002 were linked to the Taiwan Statistics of Causes of Death and National Health Insurance Research Database (NHIRD) until December 31, 2015 and were obtained from a universal, single-payer, and compulsory health insurance system that covers 99% of the 23 million residents of Taiwan, with diseases identified according to the International Classification of Disease-9 and 10 (ICD-9 and ICD-10) codes. All eligible participants in this study were excluded if they were recruited before the enrollment date of the 2002 Taiwan’s Triple High Survey and if they: (1) were aged < 20 years; (2) had a pregnancy within the previous 1 year; (3) had a recorded history of coronary artery disease and ischemic stroke in the National Health Insurance system; and (4) had missing data for identical numbers linked to the Taiwan National Health Interview Survey or National Health Insurance Research Database. A total of 6048 participants were included in the final analysis dataset used for the current analyses. Informed consent was obtained from each participant on 2002. The protocol was reviewed and approved by the Research Ethics Committee of National Taiwan University Hospital.

### Assessment of healthy lifestyle factors

The BMI was calculated as the weight in kilograms divided by the square of height in meters from self-reported data in 2002 and participants were categorized as underweight (BMI < 18.5), normal weight (18.5 ≤ BMI < 25), obesity I (25 ≤ BMI < 30), obesity II (30 ≤ BMI < 35), obesity III (35 ≤ BMI < 40), and obesity IV (BMI ≥ 40) according to the recommendations of the World Health Organization.

Data used to generate the healthy diet patterns were derived from a simplified food frequency questionnaire with 20 items of food. We used the alternative Mediterranean diet score as our healthy dietary score (supplement), which was calculated by the frequency of intake; scores across all 11 components of the 17 primary criteria contained in the Mediterranean dietary score were added for the following 39 items: fresh vegetables, legumes, fresh fruits, dairy products (milk, goat's milk, fermented milk, cheese, yogurt, or Yakult), grains (rice or noodle), meat (beef, pork, goat, or chicken), fish, eggs, sweets (cookies, candies, chocolate, cakes, bread, ice cream, or milkshake), nonalcoholic beverages (cola, soda, or sweet-beverage), and saturated lipid (burger, French frizzed, or pizza). Participants were further classified according to different levels of the alternative Mediterranean diet score (0–3, 4–5, 6–7, and 8–11 points). Participants with an alternative Mediterranean healthy diet score of ≥ 6 were assigned to the adherence of healthy diet group, whereas those with scores of < 6 were assigned to the nonadherence of healthy diet group. Physical activity during the past 2 weeks was categorized as adequate activity (1–50, 51–100, and 101–150 min/week) and nonoptimal physical activity, including inactive (0 min/week) or overactive (> 150 min/week) grading (Supplemental Table 3). Smoking status was categorized as current smoking ≥ 20 years, current smoking < 20 years, quit smoking < 1 year, quit smoking ≥ 1 year, and never smoking (Supplemental Table 4). The participants were questioned about the usually drinking status and categorized as having frequency (dinking every day with undrunk, half-drunk, or drunk status; drinking per 2 days with half-drunk or drunk status; and drinking once a week, with drunk status), less (drinking less than once a week or drinking per 2 days, with an undrunk status), or no alcohol consumption (Supplemental Table 5). A detailed description of the questions and definition of ideal BMI, healthy diet, adequate physical activity, non-smoking status, and frequency of alcohol consumption is presented in Supplemental Table 6 based on the current literature, recommended guidelines, and levels realistically obtainable within the general population.

### Healthy lifestyle scores

We created a simple pragmatic combined healthy lifestyle score to sum each dichotomous lifestyle factor as "optimal" versus "nonoptimal" as follows: normal BMI (BMI < 25 kg/m^2^) versus obese (BMI ≥ 25 kg/m^2^), alternative Mediterranean diet ≥ 6 points versus < 6 points, adequate physical activity (1–150 min/week) versus non-optimal physical activity (0 or > 150 min/week), never smoking versus current or quit smoking, and frequent drinking versus less or no drinking. The participants received 1 point for each optimal criterion met, and points were summed to obtain an MHL score ranging from 0 (nonoptimal) to 5 (optimal). A weighted MHL score was also created, where each dichotomous lifestyle factor was first weighted according to its independent magnitude of effect (e.g., beta coefficient adjusted for the other dichotomized lifestyle factors) on CVD risk ranging from 0 to 17.

In accordance with the WCRF/AICR 2018 definition, the WCRF/AICR lifestyle score was created, which was a composite numerical measure of the adherence of health lifestyle comprising seven main components, with each score based on a 0, 0.25, 0.5, and 1 scale representative from least to most healthy. We defined the WCRF/ACIR healthy lifestyle score as the sum of scores across of all seven main components, including healthy weight, physically active, a diet rich in wholegrains, vegetables, fruit, and beans, limiting consumption of “fast foods,” red and processed meat, sugar-sweetened drinks, and alcohol. Based on the 2019 AHA update criteria of cardiovascular health, the Life's Simple 7 in our study included four core health behaviors (BMI, healthy diet (Supplemental Table 9), physical activity, and non-smoking) and three health factors (cholesterol, blood pressure, and glycemic control) (Supplemental Table 10). Each healthy heart behavior and factors providing 2 points for an ideal metric, 1 point for an intermediate metric, and 0 points for a poor metric were added to obtain the Life's Simple 7 score, ranging from 0 to 14. The comparison of lifestyle components among the four healthy lifestyle scores were demonstrated in Supplement Table 11.

### Important covariates

At baseline, participants reported on sociodemographic factors and medical history, including educational levels, monthly income, marital status, menopause status, history of estrogen exposure, and parental history of CVD. Additionally, a history of the diagnosis of diabetes mellitus, hypertension, and hyperlipidemia at baseline was obtained based on the measurement in 2002 or by the ICD-9 codes or prescription of drugs from NHIRD before the enrollment date. Diabetes at baseline was defined as fasting serum glucose ≥ 126 mg/dL and hemoglobin A1c ≥ 6.5 mg/dL or records with two consistent diagnoses of diabetes by the ICD-9 codes or prescription of antidiabetic drugs for over 28 days in the data from the NHIRD before the enrollment date. Hypertension was defined as systolic blood pressure ≥ 140 mmHg or diastolic blood pressure ≥ 90 mmHg or records with two repeated diagnoses of hypertension or prescription of anti-hypertensive drugs for over 28 days in the data obtained from the NHIRD before the enrollment date. Data on the use of lipid-lowering agent and aspirin were obtained from the drug register and defined as yes when prescriptions of more than 28 days before the enrollment date were included. Abdominal obesity was indicated as a waist circumference ≥ 80 cm in women and ≥ 90 cm in men. The adjusted factors included systolic blood pressure, diastolic blood pressure, and serum biomarkers obtained during the 2002 interview. The biomarkers comprised fasting glucose, glycated hemoglobin, triglyceride, and non-high-density lipoprotein cholesterol (HDL) as continuous variables.

### Outcome ascertainment and prospective follow-up

Follow-up information was obtained from the NHIRD and Taiwan Cause of Death Register for fatal outcomes by record linkage using the personal identification numbers assigned to every citizen in Taiwan. The ICD-9 codes were used to identify CVD, such as coronary artery disease (ICD-9 codes 410–411, 414 and V45.81–82) or ischemic stroke (ICD-9 codes 433–436, 4371, 4379), with the first hospitalization with a diagnosis of the abovementioned interest events and event date defined as the first date of hospitalization (Supplemental Table 12). We ascertained the occurrence of coronary artery disease- and stroke-related deaths from the death certificate. All participants were flagged for death at the Department of Household Registration, with coded death certificates using the ICD-9 codes. The diagnoses of coronary artery disease and ischemic stroke were made by the treating physicians based on relevant clinical assessments and examinations by the clinician in charge of treatment.

### Statistical analyses

Participants were categorized into four groups among each healthy lifestyle scores (the simple and weighted MHL scores, WCRF/AICR healthy lifestyle score, and Life's Simple 7 score) (Supplemental Table 11). The continuous variables are presented as means, standard deviations (SDs), or median levels; categorical data are presented in a contingency table with analysis of variance to test for differences among quintiles. For clarifying the correlation between the simple and weighted MHL scores, WCRF/AICR lifestyle score and Life’s Simple 7, we conducted Spearman's rank correlation analysis among the four scores with a crude model adjusted for age and sex. Considering the four divided groups among the four healthy lifestyle scores with different cut-off points, we also performed the kappa(κ) analysis to evaluate the hazard ratio (HR) robust among four scores with different numbers of each group.

Multivariate Cox regression models were constructed for the combined health lifestyle scores, with the lowest score category used as the reference category (Supplemental Table 13). The linear trend test for the lifestyle scores was performed by treating the number of low-risk factors as continuous variables. The population attributable risk (PAR) was estimated using HRs obtained from the different Cox regression models in our cohort with that in the fully adjusted model. We tested potential effect modifiers based on the age category (< 60 and ≥ 60 years) using the likelihood ratio test to compare models with and without a cross-product term. Furthermore, we conducted several sensitive tests to explore the unmeasured confounding and to understand whether all 95% confidence intervals (CIs) in the primary and sensitivity analyses were reported based on robust SEs. First, we analyzed the CVD risk between both the simple and weighted MHL scores without alcohol intake because the alcohol intake was inconsistent among previous lifestyle scores. Second, we substituted BMI by waist circumflex to calculate both the simple and weighted MHL scores and to assess their association with CVD risk. Finally, we adapted each lifestyle as a categorical factor rather than dichotomous one to calculated the weighted MHL score for preventive misclassification of exposure variables and exam the association robust.

To predict the role of a combination of healthy lifestyle factors on CVD risk, we compared the four healthy lifestyle scores with traditional models, which contained clinical risk factors and all other covariates except for lifestyle behavior. We tested the prediction performance using the calibration and discrimination abilities based on the Hosmer–Lemeshow statistic^41^, comparisons of the Harrell C-index of survival data^42–44^, calibration curves, the net reclassification improvement (NRI), and integrated discrimination improvement (IDI) statistic^45^. All statistical tests were two-tailed with a type I error. Statistical significance was considered as a two-sided *p*-value of < 0.05. The SAS version 9.4 (SAS Institute, Cary, NC, USA) and Stata version 12 (Stata Corporation, College Station, TX, USA) were used for statistical analysis.

### Ethics approval and consent to participate

Informed consent had been obtained from each participant on 2002. On 2019 when we conducted the analysis, informed consent of the study participants was not required again because the dataset used in this study consists of de-identified secondary data released for research purposes. This study was conducted in accordance with the Declaration of Helsinki and was approved by the Ethical Review Board of National Taiwan University Hospital (201901103 W).

## Results

The final analytic sample included data obtained during a mean follow-up of 12.5 years, with 520 (8.6%) new cases of cardiovascular events and 20 confirmed CVD-related deaths (3.8%) that occurred during the study (Supplemental Fig. 1). The baseline characteristics of the study participants (3012 men [49.8%], 3036 women [50.2%]) included a mean (SD) age of the population at baseline of 44.9 ± 16 years, whereas the mean age at CVD diagnosis was 63.0 ± 12.8 years. The general baseline characteristics according to the number of healthy lifestyles in the simple and weighted MHL scores, WCRF/AICR lifestyle score, and Life's Simple 7 score are presented in Table 1 and Supplemental Table 14.

**Table 1:** Basic characteristics of the study participants at baseline, specified by adherence numbers of healthy lifestyle scores according to the simple Taiwan healthy lifestyle score (0 ~ 5 points).

The numbers of score	Whole population	Simple Taiwan healthy lifestyle score (%)				*P*
	(n = 6048)	0–1 (n = 1332)	2 (n = 2438)	3 (n = 1811)	4–5 (n = 461)	
Women	50.2	28.2	53.3	59.3	62	< 0.001
**Age (years) 20–39**	41.1	30.3	35.5	51.1	62.5	< 0.001
40–59	39.2	44.1	41.4	34.7	31	
≥ 60	19.7	25.6	23.2	14.2	6.5	
**Body mass index (kg/m**^**2**^**) < 25**	73.2	31.38	75.88	94.76		< 0.001
≥ 25	26.9	68.62	24.12	5.24		
**Mediterranean diet score ≥ 6**	47.2	12.84	33.96	81.47		< 0.001
< 6	52.8	87.16	66.04	18.53		
**Exercise time 1–150 min/week**	23	3.2	11.4	34.5	96.1	< 0.001
0 or > 150 min/week	77	96.8	88.6	65.5	3.9	
Never smoking	71.3	34.2	73	89.8	97	< 0.001
Quit and current smoking	28.7	65.8	27	10.2	3	
Adequate drinking	5.1	2.4	5.7	5.4	8.7	< 0.001
Non or few drinking	94.9	97.6	94.3	94.6	91.3	
**Marital status: living with spouse**	64.6	29.1	33.3	40.5	44.7	< 0.001
Single, divorced or separated	35.4	71	66.7	59.5	55.3	
**Education level: ≤ 9 years**	45.7	58.6	51	35.1	22.1	< 0.001
> 9 years	54.3	41.4	49	64.9	77.9	
**Monthly income < 40,000 NTD**	79.6	79.2	81.9	78.1	74.8	0.001
≥ 40,000 NTD	20.4	20.8	18.1	21.9	25.2	
Parents history of CVD	21.9	24.5	22.4	20.8	16.9	0.004
Menopause status	17.3	16	21.9	14.6	7.4	< 0.001
Hypertension	15.7	24.9	17.1	9.8	5.4	< 0.001
Diabetes mellitus	4.4	7.4	4.6	2.8	0.7	< 0.001
History of hyperlipidemia	7.2	9.3	7.6	5.9	4.3	< 0.001
HRT use	8	6.5	8.7	8.2	7.6	0.125

Variable	Mean (SD)	Mean				*P*
Systolic BP, mmHg	116.5 (18.2)	122.1	118	112.6	107.7	< 0.001
Diastolic BP, mmHg	75.6 (11.4)	79.6	75.9	73.3	70.9	< 0.001
Total cholesterol, mg/dL	186.1 (37.9)	191.3	187.2	182.6	178.7	< 0.001
Triglyceride, mg/dL	130.1 (86.6)	163.6	129.5	112.8	105.2	< 0.001
HDL-cholesterol, mg/dL	55.5 (15.3)	51.3	55.9	57.5	57.9	< 0.001
LDL-cholesterol, mg/dL	117.1 (27.2)	121.2	118.4	113.9	110.6	< 0.001
Non-HDL-cholesterol, mg/dL	130.6 (35.3)	140	131.3	125.2	120.8	< 0.001
Fasting glucose, mg/dL	95 (29.4)	101.1	95.5	91.8	87.1	< 0.001
Hemoglobin A1c, %	5.4 (1.1)	5.6	5.4	5.2	5.1	< 0.001

*MHL* Mediterranean diet related healthy lifestyle score, *SD* standard deviation, *BP* blood pressure, *HDL* high density lipoprotein, *LDL* low density lipoprotein, ANOVA and the chi-square tests were used to compare the means and proportions among groups.

The Spearman’s rank correlation coefficients for the four lifestyle scores ranged from 0.04 to 0.84 and 0.07 to 0.86 in the crude and adjusted models, respectively (Supplemental Table 15). The exploration demonstrated that the four scores had a monotonic relationship with each other and that there was a measured concordance of the ranks within them. Agreement among these four healthy lifestyle scores by κ values and the percentage change of κ by deleting one lifestyle score are presented in Supplemental Table 16. Among the four lifestyle scores, the simple and weighted MHL scores and Life’s Simple 7 had higher κ values than the WCRF/AICR lifestyle score (0.6 ~ 0.7 versus − 0.05), indicating higher consistency in the other three scores than in the WCRF/AICR lifestyle score. The range of percentage change of κ was around 10–20%, without large influence of the lifestyle scores.

**Figure 1:**
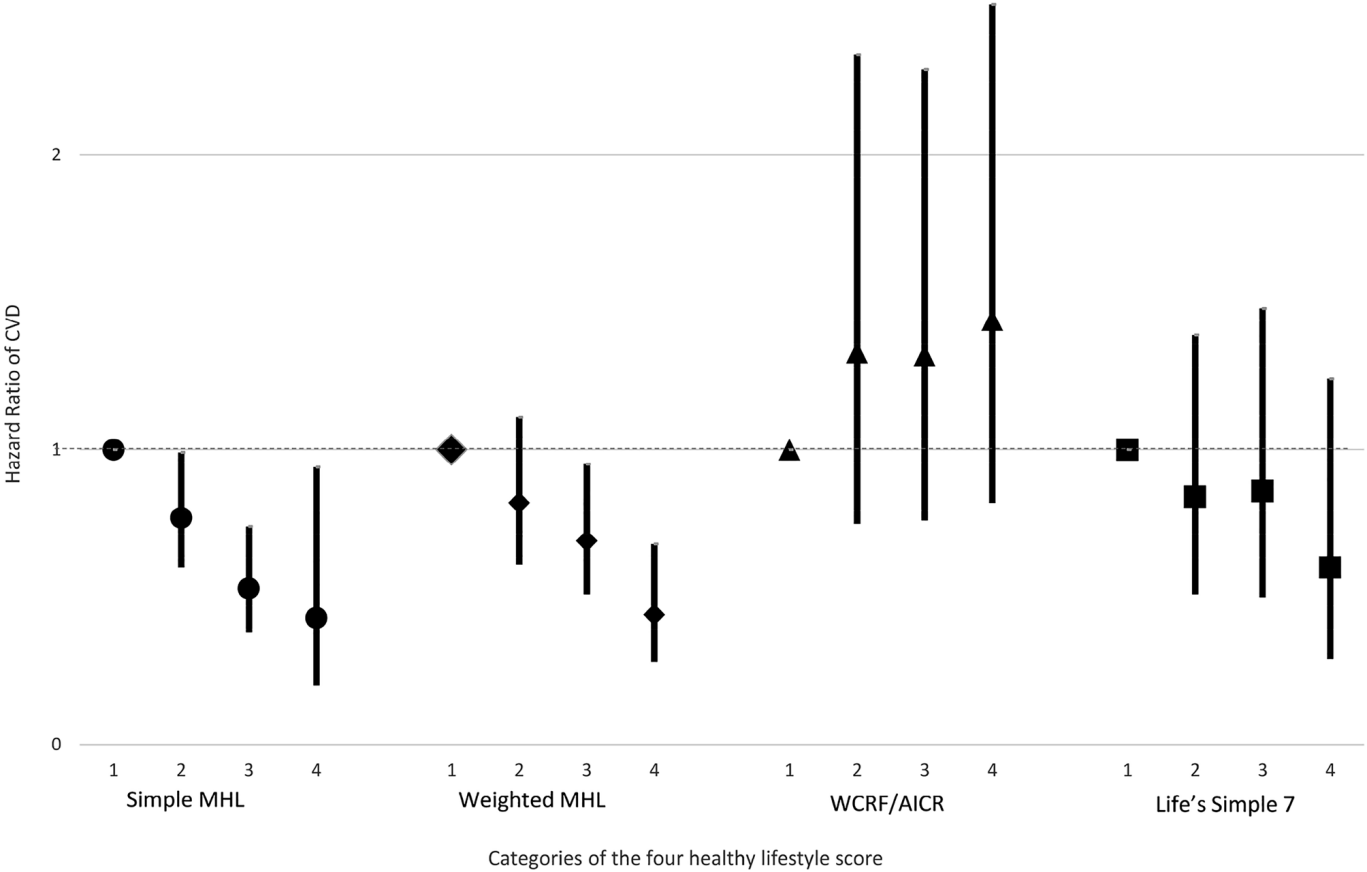
Hazard ratios for cardiovascular disease specified by categories according to the numbers of healthy lifestyle factors among participants stratified by simple Taiwan healthy lifestyle score, weighted Taiwan healthy lifestyle score, WCRF/AICR recommended healthy lifestyle score and Life’s Simple 7. *MHL* Mediterranean diet related healthy lifestyle score, *WCRF/AICR* World Cancer Research Fund/American Institute for Cancer Research.

Each lifestyle factor and CVD risk after multivariable adjustments among each lifestyle factor and the partial population attributable fraction (95% CI) are shown in Supplementary Table 15. The participants with the highest healthy lifestyle scores had a significantly higher survival rate; they were free from the CVD risk in the simple and weight MHL scores and Life's Simple 7, although this benefit was not observed for the WCRF/AICR lifestyle score (Table 2, Supplemental Table 16, and Supplemental Fig. 2). In the multivariable-adjusted analyses, adults with adherence to the highest number of lifestyle factors compared with those who adhered to the lowest numbers had a lower incidence of CVD events based on the simple (hazard ratio [HR] 0.43, 95% CI 0.2–0.94) and weighted MHL scores (HR 0.44, 95% CI 0.28–0.68), and Life’s Simple 7 (HR 0.60, 95% CI 0.29–1.24); moreover, a similar association was observed in the *p* for trend test (Table 2, Fig. 1, and Supplemental Fig. 3). However, no inverse and graded associations were noted between the WCRF/AICR lifestyle score and CVD risk, both in the Cox regression analysis and *p* for trend test. The PAR (95% CI) for participants with higher healthy lifestyle scores was estimated to be 38.8 (19.2–53.6), 34.3 (17.8–47.4), and 24.5 (3.1–41.2) for the simple MHL score, weighted MHL score, and Life's Simple 7 score, respectively. These findings suggested that the majority of CVDs may be preventable with adherence to a healthy lifestyle.

**Table 2:** The incidence cases, follow-up person-years, and rates of cardiovascular disease events according to lifestyle factors, hazard ratios, and 95% confidence intervals specified by the simple Taiwan healthy lifestyle score, according to the numbers of the score.

	O ~ 1	2			3			4 ~ 5			*P* of Logrank			
Cases	193	228			87			12						
Pearson-year	15,840.3	30,091.5			23,459.7			6163.6						
Rates/1000 py	12.18	7.6			3.71			1.95			< 0.001			

	HR	HR	95% CI		HR	95% CI		HR	95% CI		*P* of Trend Test	PAF	95% CI	
Univariate	1.00	0.56	0.45	0.69	0.26	0.20	0.35	0.14	0.07	0.27	< 0.001			
Model 1	1.00	0.72	0.58	0.90	0.48	0.35	0.64	0.37	0.19	0.72	< 0.001			
Model 2	1.00	0.75	0.60	0.94	0.53	0.39	0.71	0.42	0.21	0.82	< 0.001			
Model 3	1.00	0.77	0.60	0.99	0.53	0.38	0.74	0.43	0.20	0.94	< 0.001	38.8	19.2	53.6

Model 1: adjusted for age and sex; Model 2: Model 1, additionally education, average month income, marital status, parental history of CVD, menopause status and estrogen exposure; Model 3: Model 2 + baseline HTN, baseline DM, history of hyperlipidemia, sBP, dBP, triglyceride, non-HDL, fasting glucose, HbA1c; The population attributable risk is the percentage of new cases of heart failure in the population attributable to nonadherence to the low-risk lifestyle factor.

When we stratified the study population by age at baseline (< 60 or ≥ 60 years), a significantly inverse association between the healthy lifestyle scores and CVD risk for a given number of lifestyle factors in all four healthy lifestyle scores (Table 3) persisted, although participants aged < 60 years had a greater reduction of CVD risk than those aged ≥ 60 years (Supplemental Fig. 4). Thus, the analyses indicated that the protective effect of the healthy lifestyle scores for CVD incidence indeed varied by age in adult participants. In sensitivity analysis, no matter the calculation of simple and weighted MHL scores with or without alcohol intake factor, substitute of body mass index by waist circumflex or sum of the weighted MHL score by categorical variable than dichotomous ones, all sensitivity analyses reported the preventive CVD risk effect from both lifestyle score persistently (Supplemental Table 17) and no unobserved confounders were detected.

**Table 3:** Hazard ratios for cardiovascular disease among participants stratified by age < 60 years/o and ≥ 60 years/o, specified by simple Taiwan healthy lifestyle score, weighted Taiwan healthy lifestyle score, WCRF/AICR recommended healthy lifestyle and Life’s Simple 7 according to the numbers of healthy lifestyle factors.

	Group 1	Group 2			Group 3			Group 4			P_interaction_
	HR	HR	95% CI		HR	95% CI		HR	95% CI		
**Simple MHL**											
Age < 60	1	0.65	0.44	0.96	0.37	0.21	0.63	0.35	0.13	0.99	0.070
Age ≥ 60	1	0.88	0.64	1.22	0.67	0.44	1.03	0.56	0.17	1.79	
**Weighted MHL**											
Age < 60	1	0.60	0.37	0.96	0.53	0.32	0.88	0.31	0.15	0.61	0.029
Age ≥ 60	1	0.96	0.66	1.41	0.81	0.54	1.21	0.60	0.33	1.07	
**WCRF**											
Age < 60	1	0.86	0.45	1.64	0.84	0.44	1.59	1.30	0.67	2.51	0.011
Age ≥ 60	1	3.26	0.77	13.84	2.95	0.71	12.26	2.65	0.64	11.04	
**LS7**											
Age < 60	1	1.25	0.59	2.63	0.61	1.24	0.54	0.97	0.32	2.98	0.234
Age ≥ 60	1	0.62	0.31	1.23	0.26	0.66	0.32	0.45	0.17	1.18	

Group 1 as those with lowest number of healthy lifestyle score, Group 2 and Group 4 as those with increasing the numbers of healthy lifestyle score.

### Assessment of model performance of four healthy lifestyle scores

The Hosmer–Lemeshow test statistic indicated an acceptable goodness-of-fit of the calibration ability, and the model was well calibrated for 12.5-year CVD risk prediction based on the calibration in four healthy lifestyle scores (Table 4 and Supplemental Fig. 5). With regard to the discriminative ability of different healthy lifestyle scores to predict the CVD risk, the Harrell C-statistics significantly increased from 0.85 to 0.86 (*p*_diff_ = 0.02) for the simple MHL score and it significantly increased from 0.84 to 0.87 (*p*_diff_ = 0.003) for the weighted MHL score (Supplemental Fig. 6). Moreover, we found that the performance measures evaluated by the IDI showed a significant improvement of 0.38% (95% CI; 0.01, 0.74; *p* = 0.021) for the simple MHL score and 0.51% (95% CI; 0.16, 0.86; *p* = 0.002) for the MHL score. The NRI was statistically significant for the Mediterranean diet-related healthy lifestyle score (0.03; 95% CI 0.01–0.05; *p* = 0.004) and MHL score (0.04; 95% CI 0.02–0.06; *p* < 0.001). However, the Harrell C-statistics, IDI, and NRI showed no significant difference for the WCRF/AICR healthy lifestyle score or Life’s Simple 7 score.

**Table 4:** Improvement in discrimination performance and calibration for risk prediction of cardiovascular events in the multivariate-adjusted model after including simple Mediterranean diet related healthy lifestyle (MHL) score, weighted MHL score, WCRF/AICR recommendation lifestyle and Life's Simple 7.

	AUC	95% CI		P	P for HL test	IDI (%)	95% CI		P	NRI (%)	95% CI		P
Clssical model	0.85	0.837	0.870	Reference									
Simple MHL	0.86	0.842	0.874	0.02	0.34	0.38	0.01	0.74	0.021	0.03	0.01	0.05	0.004
Weighted MHL	0.86	0.840	0.873	0.003	0.25	0.51	0.16	0.86	0.002	0.04	0.02	0.06	< .0.001
WCRF/AICR	0.85	0.838	0.870	0.49	0.49	0.10	− 0.03	0.24	0.07	0.07	− 1.15	1.29	0.91
Life's Simple 7	0.85	0.837	0.870	0.80	0.73	0.09	− 0.06	0.24	0.11	0.95	− 0.37	2.28	0.16

CVD risk classification (0, 0.01, 0.05, 0.1).

## Discussion

In this representative adult Taiwanese study population, 38.8% of all CVD events may have been avoided had all participants adhered to a healthy lifestyle with regard to normal weight, healthy Mediterranean diet, regular physical activity, non-smoking, and adequate healthy drinking. Moreover, we noted the protective effect of the simple and weighted MHL scores and Life’s Simple 7 score for CVD risk reduction. Furthermore, we noted that the age of adult participants had a modifier effect on the inverse association between the healthy lifestyle scores and CVD risk. Younger and hypertension-free participants who adopted an optimal lifestyle derived greater benefits than the older adult population.

The findings of this study are consistent with those of previous cohort studies in European, American, and Asian populations and suggest a protective effect of the healthy lifestyle scores and CVD risk in an extensive Chinese general population. However, previous cohort studies had limitations with regard to the analysis of adjusted covariates, including age, sex, socioeconomic status, parental history of CVD, and medical history at baseline, although a few studies adjusted the analysis for clinical factors^7,9,16,17,22–24,26^. In this study, although aspirin and lipid-lowering agents were not included in our adjusted models because of their low prevalence among the study population at baseline, we estimated the HR after adjusting the models for age, sex, socioeconomic, and health status at baseline and for clinical factors, such as blood pressure, fasting glucose levels, triglyceride levels, and non-HDL cholesterol levels. All of these results from studies including clinical factors as adjustment covariates imply that the combined lifestyle interventions had an additional benefit for decreasing the CVD incidence by mechanisms other than those associated with controlling blood pressure, glucose, and lipid levels.

Upon comparing the weighted lifestyle score and simple lifestyle score to examine the assumption of each lifestyle factor with the same magnitude effect of the CVD risk by the area under the curve, the IDI and NRI demonstrated similar predictive performance for CVD incidence. This result was consistent with those from previous studies on a healthy lifestyle and risk of heart failure^6^, with relevance as the first study of the weighted healthy lifestyle score and CVD risk. The similar results for both lifestyle scores might imply that there was no additional benefit of focusing on a single or two healthy behaviors than that of the integration of all healthy lifestyle factors. Moreover, adopting an overall healthy lifestyle rather than a strong emphasis on a particular lifestyle was an optimal strategy to improve cardiovascular health.

Multiple observational studies have reported an inverse association between adherence to a high WCRF/AICR lifestyle score and various cancer incidences^46–49^. Previous studies on the association between greater adherence to the WCRF/AICR lifestyle score and the CVD risk factors were limited and they yielded inconsistent findings. A cross-sectional study reported that an increasing adherence to the WCRF/AICR recommendation decreased the incidence of metabolic syndrome^50^, whereas another study observed that it was associated with higher serum levels of thrombomodulin and thrombopoietin that might increase the CVD risk^51^. To the best of our knowledge, this is the first prospective cohort study that reported the association between the WCRF/AICR lifestyle score and CVD risk, although the analyses demonstrated nonsignificant associations.

The inverse linear association between Life's Simple 7 and short-term and lifetime risk of CVD and all-cause mortality, which would maintain for several decades after all index ages, has been confirmed in repeated re-analyses^52–63^. Our finding showing that participants with high adherence to Life's Simple 7 had 40% decreased risk of CVD compared with those with poor adherence is consistent with previous reports. However, in our study wherein several clinical risk factors and biomarkers were adjusted, the cardiovascular protection conferred from a higher Life's Simple 7 score was attenuated. This decline in the preventive effect against CVD incidence conferred from the Life's Simple 7 score implies that the protective benefits are derived more from the clinical risk factors than from lifestyle factors, which drive the score. This shifts the focus from primordial prevention to primary prevention of clinical risk factors and toward the targeting of individuals with higher short-term, rather than a long-term, CVD risk.

Compared with different healthy lifestyle scores, the MHL score might be more suitable for primordial prevention in populations without clinical risk factors. The different CVD risk performances might be explained by the different definitions of healthy diet, physical activity, and the lack of alcohol consumption or smoking. The Mediterranean diet evaluated in the MHL score comprised fish as an optimal food for CVD protection but with limited egg and dairy in the daily dietary intake. Moreover, the MHL score defined regular adequate alcohol consumption as a part of an optimal lifestyle. However, the WCRF/AICR lifestyle score considers no alcohol intake to be an ideal lifestyle, whereas the Life's Simple 7 does not take the amount of alcohol consumption into consideration. Additionally, the non-smoker or cessation of smoking status contributed to the MHL score and Life's Simple 7 score, although it was not calculated in the WCRF/AICR lifestyle score.

Multiple reasons (observed in this study) might explain the benefits derived from a healthy lifestyle in younger populations than in the elderly. First, aging is an original strong risk factor for atherosclerosis. Research has reported that even in individuals with an ideal modifiable lifestyle and healthy status, a high CVD risk still persists among people aged 65–75 years depending on different racial influences^64^. Approximately 60% of the 10-year predicted atherosclerotic CVD risk was attributable to age alone. Additionally, the magnitude of causal association between lifestyle and CVD risk might be reduced when age is included as a significant covariate^65^. Second, a legacy effect of CVD risk from nonoptimal behavioral factors leading to a persistent pathological change even in individuals who have an optimal lifestyle has been reported recently^66^. Older people have a longer lifetime to experience a nonoptimal lifestyle than younger individuals, and the legacy effect of nonoptimal lifestyle behaviors should be considered. Finally, people with chronic diseases would have a stronger motivation to maintain a healthy lifestyle. However, the higher prevalence of chronic diseases among the elderly may play the role of a potential confounder in CVD incidence, reducing the protective effect against CVD obtained from a healthy lifestyle.

The clinical implications include the identification of unhealthy lifestyle factors among young and middle-aged adults; aggressive healthy lifestyle interventions are crucial for improving the population’s cardiovascular health. Additionally, among populations with low short-term risks, healthy lifestyle scores in the absence of clinical risk factors independently provided additional important information about the long-term CVD risk and overall CVD burden. Furthermore, the simple MHL score, as well as the weighted score, was a useful tool in primary health services, even at home and without the clinical setting, which may broaden public health screening without the need for laboratory-based measures; this may have implications for the development of health policies with different strategies from that of the primordial and primary prevention of CVD.

This study has several strengths. The TWsHHH evaluated a population of middle-aged adults with low prevalence of clinical risk factors in a national representative cohort and little loss to follow-up over 12.5 years; information was obtained on the primary lifestyle habits and direct effect measures of clinical risk factors and biomarkers. Second, protection from the healthy lifestyle score on the CVD risk was validated in an Asian population as a primordial preventive policy in response to the 2025 global target of the World Health Organization among Western countries that extends to the Asian population. Additionally, to the best of our knowledge, this is the first study that estimated the CVD risk specifically from the WCRF/AICR recommended lifestyle score and compared predictive performance for CVD risk among different healthy lifestyle scores, including biomarkers. Finally, age as an effect modifier between the association was demonstrated by the primary data validation.

Nonetheless, some study limitations should be mentioned. The MHL score was assessed in the TWsHHH cohort without external data validation. Additionally, lifestyle factors recorded at the baseline without repeated assessments confer a potential for non-differential misclassification in the study. Nevertheless, if the association between the healthy lifestyle score and CVD risk was significant with regard to a misclassification bias, the true relative risk among these factors should be more effective due to the subsequent repeated measurements of exposure.

Finally, although the Mediterranean diet was demonstrated to significantly reduce CVD incidence, the Western diet score might not be suitable for the Asian dietary habit. Further CVD prediction studies in the Asian population should consider the locally diet pattern, such as Taiwanese Eating Approach scores^67^, which excluded legumes, dairy products, chicken, eggs, and sweets but included seafood (shrimp or crabs), seaweed, mushrooms, tea, fatty meats, and fermented vegetables in the scale.

## Conclusion

Adherence to a high combined healthy lifestyle score plays an important role in the primary prevention of CVD, especially in younger adults with low short-term risks. All simple and weighted MHL scores and Life's Simple 7 have effective prediction of CVD risk compared with the WCRF/AICR lifestyle score in the adult Taiwanese population. Additionally, simple and weighted MHL scores entirely established on lifestyle factors improved the primary CVD prevention to primordial prevention. Further investigation on the mechanism of CVD prevention based on healthy lifestyle scores independent of the clinical factors is necessary to validate these findings.

## Supplementary Information

Supplementary Information.

## Data Availability

The datasets generated and/or analyzed during the current study are not publicly available due to the policy declared by “H_BHP_TWHHH _Database” (https://dep.mohw.gov.tw/dos/lp-3147-113-5-20.html)” but are available from the corresponding author on reasonable request.

## Abbreviations

MHL: Mediterranean diet related healthy lifestyle score
CVD: Cardiovascular disease
BMI: Body mass index
WCRF/AICR: World Cancer Research Fund and the American Institute for Cancer Research
NHIRD: National Health Insurance Research Database
HR: Hazard ratios
IDI: Integrated discriminative improvement
NRI: Net reclassification improvement
