# Supplementary material for: Comparison of four healthy lifestyle scores for predicting cardiovascular events in a national cohort study
Source: Sci Rep. 2021 Nov 12;11:22146. doi: 10.1038/s41598-021-01213-6 (PMC8589956; doi:10.1038/s41598-021-01213-6)
Supplement: Supplementary file 1 — Supplementary Information. [file 41598_2021_1213_MOESM1_ESM.docx]

**Supplement**

[Supplemental Table 1: The official and our study definition of body mass index (BMI) 3](#_Toc83291932)

[Supplemental Table 2: The official and our study definition of alternative Mediterranean Diet 4](#_Toc83291933)

[Supplemental Table 3: The category and definition of physical activity 6](#_Toc83291934)

[Supplemental Table 4: The category and definition of smoking status 7](#_Toc83291935)

[Supplemental Table 5: The category and definition of adequate alcohol consumption 8](#_Toc83291936)

[Supplemental Table 6: The components of simple Mediterranean diet related healthy lifestyle score 9](#_Toc83291937)

[Supplemental Table 7: The components of weighted Mediterranean diet related healthy lifestyle score 10](#_Toc83291938)

[Supplemental Table 8: The official 2018 WCRF/AICR recommended healthy lifestyle score and our study definition 11](#_Toc83291939)

[Supplemental Table 9: Life’s Simple 7 healthy diet criteria: reference from 2010 AHA definition of ideal Cardiovascular Health and our study definition 14](#_Toc83291940)

[Supplemental Table 10: Life’s Simple 7 criteria: reference from 2010 AHA definition of ideal Cardiovascular Health and our study definition 15](#_Toc83291941)

[Supplemental Table 11: 17](#_Toc83291942)

[Supplemental Table 12: International Classification of Diseases—Clinical Modification (ICD-CM) and procedure codes used to define the metabolic syndrome and clinical outcome in the study cohort 19](#_Toc83291943)

[Supplemental Table 13: The definition of important covariates and multivariate COX regression model 22](#_Toc83291944)

[Supplemental Table 14: Basic characteristics of the study participants at baseline, specified by adherence numbers of healthy lifestyle scores; (A). The weighted Mediterranean diet related healthy lifestyle score (0~17 points) (B) The WCRF/AICR recommended healthy lifestyle score (0~7 points) (C) Life’s Simple 7 score (0~14 points) 24](#_Toc83291945)

[Supplemental Table 15: The Spearman’s correlation coefficients between the four healthy lifestyle scores; (A) crude model (B) model with adjusted age and sex; MHL: Mediterranean diet related healthy lifestyle score; WCRF/AICR: World Cancer Research Fund/American Institute for Cancer Research 27](#_Toc83291946)

[Supplemental Table 16 : Interrater agreement of different criteria of healthy lifestyle score by κ values (A), each cell presenting the κ when deleting this criterion (B); MHL: Mediterranean diet related healthy lifestyle score; WCRF/AICR: World Cancer Research Fund/American Institute for Cancer Research 29](#_Toc83291947)

[Supplemental Table 17: The incidence cases, follow-up person-years, and the rates of cardiovascular disease events according to lifestyle factors and the hazard ratios and 95% confidence intervals 31](#_Toc83291948)

[Supplemental Table 18: The incidence cases, follow-up person-years, and the rates of cardiovascular disease events according to lifestyle factors and the hazard ratios and 95% confidence intervals; (A) The weighted MHL healthy lifestyle score, according to the score quartiles (B) The WCRF/AICR recommended healthy lifestyle score, according to the numbers of the score (C) Life’s Simple 7, according to the numbers of score (0~14 points); MHL: Mediterranean diet related healthy lifestyle score; WCRF/AICR: World Cancer Research Fund/American Institute for Cancer Research 37](#_Toc83291949)

[Supplemental Table 19: Sensitivity test of the incidence cases, follow-up person-years, and the rates of cardiovascular disease events and the hazard ratios and 95% confidence intervals 40](#_Toc83291950)

[Supplemental Figure 1: Flow-chart of study design and participants exclusion criteria 45](#_Toc83292127)

[Supplemental Figure 2: Kaplan-Meir survival curves for the risk of cardiovascular specified in the study participants among participants stratified by the numbers of healthy lifestyle factors (A) by simple Mediterranean diet related healthy lifestyle score (B) by Mediterranean diet related healthy lifestyle score (C) by WCRF/AICR recommended lifestyle score (D) by Life’s Simple 7; MHL: Mediterranean diet related healthy lifestyle score; WCRF/AICR: World Cancer Research Fund/American Institute for Cancer Research 46](#_Toc83292128)

[Supplemental Figure 3: The log(-log(survival time)) versus log of CVD event-free survival time including time independent covariates 50](#_Toc83292129)

[Supplemental Figure 4: Hazard ratios for cardiovascular disease with categories according to the numbers of healthy lifestyle factors among participants stratified by age < 60 y/o and ≥ 60 y/o 59](#_Toc83292130)

[Supplemental Figure 5: Calibration plot of predicted mean 12.5.following-up years cardiovascular disease (CVD) risk within deciles against the observed 12.5 following-up years CVD risk in the TWsHHH data (N=6048). Data are plotted among participants stratified by lifestyle scores 63](#_Toc83292131)

[Supplemental Figure 6: Receiver-operating characteristic curves for various models applied to the study population; MHL: Mediterranean diet related healthy lifestyle score; WCRF/AICR: World Cancer Research Fund/American Institute for Cancer Research 67](#_Toc83292132)

Supplemental Table 1: The official and our study definition of body mass index (BMI)

|  | WHO BMI Categories | Taiwan Healthy lifestyle scoring |
| --- | --- | --- |
| BMI <18.5 | Under weight | 1 |
| BMI>=18.5 and <25 | Normal weight | 1 |
| BMI>=25 and <30 | Obesity I | 0 |
| BMI>=30 and <35 | Obesity II | 0 |
| BMI>=35 and <40 | Obesity III | 0 |
| BMI>=40 | Obesity IV | 0 |

Supplemental Table 2: The official and our study definition of alternative Mediterranean Diet

| **Primary diet metric** | **Mediterranean Definition** | | **Our Definition** | | |
| --- | --- | --- | --- | --- | --- |
|  | 0 point | 1 point |  | 0 point | 1 point |
|  | The amount of intake | |  | The frequency of intake (time/week) | |
| Vegetables | < Median | ≥ Median | Fresh vegetables | < 7 | ≥ 7 |
| Legumes | < Median | ≥ Median | Legumes | < 1.5 | ≥ 1.5 |
| Fruits and nuts | < Median | ≥ Median | Fresh fruits | < 7 | ≥ 7 |
| Dairy products | ≥ Median | < Median | Milk, goat’s milk, fermented milk, cheese, yogurt, Yakult | ≥ 1.5 | < 1.5 |
| Cereals | < Median | ≥ Median | Rice or Noodles every day | < 2 (cup/day) | ≥ 2 (cup/day) |
| Meat | ≥ Median | < Median | Beef, pork, goat, chicken | ≥ 4 | < 4 |
| Fish | ≥ Median | < Median | Fish | < 4 | ≥ 4 |
| Olive oil | 0 | 250 |  |  |  |
| Potatoes | ≥ Median | < Median |  |  |  |
| Eggs | ≥ Median | < Median | Eggs | ≧ 4 | < 4 |
| Sweets | ≥ Median | < Median | Cookies, candies, chocolate, cakes, bread, ice cream, milkshake | Cookies, candies, chocolate, ice cream, milkshake≧0.5  cake, bread ≧1.5 | Cookies, candies, chocolate, ice cream, milkshake < 0.5  cake, bread < 1.5 |
| Nonalcoholic beverages | ≥ Median | < Median | Cola, soda or sweet-beverage | ≥ 0.5 | < 0.5 |
| Monounsaturated lipids | < Median | ≥ Median |  |  |  |
| Saturated lipids | ≥ Median | < Median | Burger, French frizzed, pizza | > 0 | = 0 |
| Polyunsaturated lipids | ≥ Median | < Median |  |  |  |
| Percentage energy from saturated lipids | ≥ Median | < Median |  |  |  |
| Ratio of monounsaturated lipids to saturated lipids | < Median | ≥ Median |  |  |  |
| Energy intake | ≥ Median | < Median |  |  |  |

Supplemental Table 3: The category and definition of physical activity

| Total time (mins/week) |  | Healthy lifestyle scoring |
| --- | --- | --- |
| 0 | Inactive | 0 |
| 1~50 | Somewhat active | 1 |
| 51~100 | Intermittent active | 1 |
| 101~150 | Active | 1 |
| > 150 | Over active | 0 |

Supplemental Table 4: The category and definition of smoking status

| Smoking status | Healthy lifestyle scoring |
| --- | --- |
| Current smoking ≧20 years | 0 |
| Current smoking < 20 years | 0 |
| Quit smoking < 1 year | 0 |
| Quit smoking ≧1 year | 0 |
| Never or smoking less than 100 cigarettes | 1 |

Supplemental Table 5: The category and definition of adequate alcohol consumption

|  | The frequency of drinking | | | | |
| --- | --- | --- | --- | --- | --- |
|  | Once / day | Once / 2-3 days | Once / week | 1-2 times / month | < 1/month |
| Light | Frequency | Few | Few | Few | Few |
| Semi-drunk | Frequency | Frequency | Few | Few | Few |
| Drunk | Frequency | Frequency | Frequency | Few | Few |

Few = 0 point; Frequency=1 points

Supplemental Table 6: The components of simple Mediterranean diet related healthy lifestyle score

|  | BMI (Kg/m^2^) | Mediterranean diet | Physical activity (min/week) | Non-smoking status | Alcohol consumption |
| --- | --- | --- | --- | --- | --- |
| Unfavorable (0 point) | ≧25 | < 6 points | 0 or > 150 | Current and former | No or few |
| Healthy (1 point) | < 25 | ≧6 points | 1~150 | Never | Frequency |

Mediterranean diet related healthy lifestyle score = BMI + Mediterranean diet + Physical activity + non-smoking status + Alcohol consumption

Supplemental Table 7: The components of weighted Mediterranean diet related healthy lifestyle score

|  | BMI (Kg/m^2^) | Mediterranean diet | Physical activity (min/week) | Non-smoking status | Alcohol consumption |
| --- | --- | --- | --- | --- | --- |
| Unfavorable (0 point) | ≧25 | < 6 points | 0 or > 150 | Current and former | No or few |
| Healthy (1 point) | < 25 | ≧6 points | 1~150 | Never | Frequency |
| Coefficient | -0.25 | -0.29 | -0.28 | -0.18 | -0.66 |

Taiwan healthy lifestyle score = 2*BMI + 3*Mediterranean diet + 3*Physical activity + 2*Non-smoking status + 7*Alcohol consumption

Supplemental Table 8: The official 2018 WCRF/AICR recommended healthy lifestyle score and our study definition

| 2018 WCRF/AICR recommendation | Operationalization of recommendation | Our definition | points |
| --- | --- | --- | --- |
| Be a healthy weight | BMI (kg/m^2^): | BMI (kg/m^2^): |  |
|  | 18.5–24.9 | 18.5–24.9 | 0.5 |
|  | 25–29.9 | 25–29.9 | 0.25 |
|  | <18.5 or ≥30 | <18.5 or ≥30 | 0 |
|  | Waist circumference (cm): | Waist circumference (cm): |  |
|  | Men: <94 Women: <80 | Men: <94 Women: <80 | 0.5 |
|  | Men: 94–<102 Women: 80–<88 | Men: 94–<102 Women: 80–<88 | 0.25 |
|  | Men: ≥102 Women: ≥88 | Men: ≥102 Women: ≥88 | 0 |
| Be physically active | Total moderate-vigorous physical activity (mins/week) | Total moderate-vigorous physical activity (mins/week) |  |
|  | ≥150 | ≥150 | 1 |
|  | 75–<150 | 75–<150 | 0.5 |
|  | <75 | <75 | 0 |
| Eat a diet rich in wholegrains, vegetables, fruit and beans | Fruits and vegetables (g/day): | The frequency of fruits and vegetables intake |  |
|  | ≥400 | One of them every day | 0.5 |
|  | 200–<400 | One of them 1~5 times/week | 0.25 |
|  | <200 | Both of them less than once/week | 0 |
|  | Total fiber (g/day): | The frequency of bean intake |  |
|  | ≥30 | Everyday | 0.5 |
|  | 15–<30 | 1~5 times per week | 0.25 |
|  | <15 | Less than 1 time/week | 0 |
| Limit consumption of “fast foods” and other processed foods high in fat, starches or sugars | Percent of total kcal from ultra-processed foods (aUPFs) | The frequency of French fried/ pizza intake |  |
|  | Tertile 1 | Tertile 1 | 1 |
|  | Tertile 2 | Tertile 2 | 0.5 |
|  | Tertile 3 | Tertile 3 | 0 |
| Limit consumption of red and processed meat | Total red meat (g/wk) and processed meat (g/wk): | The frequency of pork, beef, goat, chicken and burger intake |  |
|  | Red meat <500 and processed meat <21 | Tertile 1 | 1 |
|  | Red meat <500 and processed meat 21–<100 | Tertile 2 | 0.5 |
|  | Red meat >500 or processed meat ≥100 | Tertile 3 | 0 |
| Limit consumption of sugar-sweetened drinks | Total sugar-sweetened drinks (g/day): | The frequency of cola, soda and other sweetened drinks intake |  |
|  | 0 | No intake | 1 |
|  | >0–≤250 | Less than 3 times/week | 0.5 |
|  | >250 | 3 times or more/week | 0 |
| Limit alcohol consumption | Total ethanol (g/day): |  |  |
|  | 0 | No drinking | 1 |
|  | >0–≤28 (2 drinks) males and ≤14 (1 drink) females | Few drinking | 0.5 |
|  | >28 (2 drinks) males and >14 (1 drink) females | Frequency drinking | 0 |

Total scoring of 2018 WCRF/AICR lifestyle score: 0-7 points

| WCRF/AICR Score | < 3 | 3~ <4 | 4~ <5 | 5~7 |
| --- | --- | --- | --- | --- |
|  | Unfavorable | Less healthy | Intermediate | Healthy |

Supplemental Table 9: Life’s Simple 7 healthy diet criteria: reference from 2010 AHA definition of ideal Cardiovascular Health and our study definition

| Recommended | | Our study definition | | |
| --- | --- | --- | --- | --- |
| Metric | Optimal amount | Metric | The frequency of intake, times / week | |
|  |  |  | 0 point | 1 point |
| Fruits and vegetables | ≧ 4.5 cups per day | Fruits or vegetables | < 7 | ≧ 7 |
| Fish | ≧ 2* 3.5-oz servings per week | Fish | <1 | ≧ 1 |
| Fiber-rich whole grains (≧1.1 g of fiber per 10 g of carbohydrate) | ≧ 3*1-oz-equivalent servings per day | Rice or noodles | < 1 (cup/day) | ≧ 1 (cup/day) |
| Sodium | < 1500 mg per day |  |  |  |
| Sugar-sweetened beverages | ≦ 450 kcal (36 oz) per week. | Cola, soda, sugar-sweetened neverages | ≧ 1 | < 1 |
| Nuts, legumes, and seeds | ≧ 4 servings per week | Legumes | < 7 | ≧ 7 |
| Processed meats | ≦ 2 servings per week | Beef, pork, goat, chieken and burger | ≧ 1 | < 1 |
| Saturated fat | < 7% of total energy intake | French fried and pizza | ≧ 1 | < 1 |

Total Life’s Simple 7 healthy diet score: 0~7 points

Supplemental Table 10: Life’s Simple 7 criteria: reference from 2010 AHA definition of ideal Cardiovascular Health and our study definition

| Metric | Recommended Optimal Level  (2 points) | Intermediate Level  (1 point) | Poor Level  (0 point) |
| --- | --- | --- | --- |
| Body mass index  Recommendation | <25 | 25-29.9 | ≥30 |
| Our definition | <25 | 25-29.9 | ≥30 |
| Healthy diet: Recommendation |  |  |  |
| Our definition (below) | 5~7 | 3~4 | 0~2 |
| Physical activity: Recommendation (mins/week) | ≧150 moderate intensity or ≧75 vigorous intensity or combination | 0~149 min/week moderate intensity or 0~74 min/week vigorous intensity | 0 min/week |
| Our definition |  |  |  |
| Smoking: Recommendation | Never or quit ≥12 month prior | Quit <12 month | Current smokers |
| Our definition | Never or quit ≥12 month prior | Quit <12 month | Current smokers |
| Total cholesterol Recommendation | <200 mg/dL untreated | 200-240 mg/dL or <200 mg/dL treated | >240 mg/Dl |
| Our definition | <200 mg/dL untreated | 200-240 mg/dL | >240 mg/dL |
| Blood pressure Recommendation | <120/80 mm Hg untreated | <120/80 mm Hg treated or 120-139/80-89 mm Hg | ≥140/90 mm Hg |
| Our definition | <120/80 mm Hg untreated | 120-139/80-89 mm Hg | ≥140/90 mm Hg |
| Fasting plasma glucose Recommendation | <100 mg/dL untreated | 100-126 mg/dL or <100 mg/dL treated | >126 mg/dL |
| Our definition | <100 mg/dL untreated | 100-126 mg/dL | >126 mg/dL |

Supplemental Table 11:

The comparisons of lifestyle components (A) and divided categorical scale (B) among the four different healthy lifestyle scores; MHL: Mediterranean diet related healthy lifestyle score; WCRF/AICR: World Cancer Research Fund/American Institute for Cancer Research

(A)

|  |  |  |  |  |  |  |
| --- | --- | --- | --- | --- | --- | --- |
|  | BMI | Diet | Physical activity | Smoking status | Alcohol consumption | Other |
| Simple MHL score | Including | Alternative Mediterranean diet | Including | Including | Including | Sum of all factors |
| Weighted MHL score | Including | Alternative Mediterranean diet | Including | Including | Including | Sum of all weighted factors |
| WCRF/AICR Score | Including | WCRF/AICR diet criteria | Including | No including | Including |  |
| Life’s Simple 7 | Including | Life's simple 7 diet criteria | Including | Including | No including | Including blood pressure, total cholesterol and fasting glucose |

(B)

|  | Unfavorable | Less healthy | Intermediate | Healthy |
| --- | --- | --- | --- | --- |
| Simple MHL score (0~5) | 0~1 | 2 | 3 | 4~5 |
| Weighted MHL score (0~17) | 0~4 | 5 | 6~7 | 8~17 |
| WCRF/AICR Score | < 3 | 3~ <4 | 4~ <5 | 5~7 |
| Life’s Simple 7 (0~14) | 0~6 | 7~9 | 10~12 | 13~1 |

Supplemental Table 12: International Classification of Diseases—Clinical Modification (ICD-CM) and procedure codes used to define the metabolic syndrome and clinical outcome in the study cohort

| Diagnosis | Definition | ICD-9 code | ICD-10 code | Procedure code |
| --- | --- | --- | --- | --- |
| Hypertension | ≥2 Outpatient department | 401-405, 437.2 | I10, I11.9, I11.0, I12.9, I12.0, I13.10, I13.0, I13.11, I13.2, I15.0, I15.1, I15.2, I15.8, I15.9, N26.2, , I67.4 |  |
| Diabetes mellitus | ≥2 Outpatient department | 250 | E08, E11, E13 |  |
| Dyslipidemia | ≥2 Outpatient department | 272 | E78.5 |  |
| Coronary heart disease | Either discharge diagnosis of ICD-9 or ICD-10 or procedure code | 410 | I21.01, I21.02, I21.09, I21.11, I21.19, I22.0, I22.1 | revascularization PCI (33076B, 33077B, 33078B)  CABG(68023B, 68024B, 68025B)  N26002, N26003 |
|  |  | 411 | I20.0, I24.0, I24.1, I24.8, I24.9 |  |
|  |  | 414.00 | I25.10, I25.750, I25.751, I25.758, I25.759, I25.760, I25.761, I25. 768, I25.769, I25. 811, I25.812 |  |
|  |  | 414.01 | I25.10, I25.110, I25.111, I25.118, I25.119, I25.750, I25.751, I25.758, I25.759, I25. 811 |  |
|  |  | 414.02 | I25.710, I25.711, I25.718, I25.719, I25.812, |  |
|  |  | 414.03 | I25.730, I25.731, I25.738, I25.739 |  |
|  |  | 414.04 | I25.720, I25.721, I25.728, I25.729 |  |
|  |  | 414.05 | I25.700, I25.701, I25.708, I25.709, I25.730, I25.731, I25.738, I25.739, I25.760, I25.761, I25.768, I25.769, I25.790, I25.791, I25.798, I25.799, I25.810, I25.812 |  |
|  |  | v45.81 | Z95.1 |  |
|  |  | v45.82 | Z95.5, Z98.61, Z95.8, |  |

Supplement Table 12. (Continued)

| Diagnosis | Definition | ICD-9 | ICD-10 | Procedure code |
| --- | --- | --- | --- | --- |
| Stroke | discharge diagnosis of ICD-9 or ICD-10 | 433 | 165.1,I63.02, I63.12, I65.21, I63.22, I65.1, I65.23, I65.29 , I63.031, I63.032, I63.039, I63.131, I63.132, I63.139, I63.231, I63.232, I63.239, I65.01, I65.02, I65.03, , I65.09, I63.011, I63.012, I63.019, I65.22, I63.111, I63.112, I63.119, I63.211, I63.212, I63.219, I65.8, 163.09, I63.19, I63.59, I65.9, I63.00, 163.10, I63.20, I63.29 |  |
|  |  | 434 | I66.01, I66.02, I66.03, I66.09, I66.11, I66.12, I66.13, I66.19, I66.21, I66.22, I66.23, I66.29, I66.3, I63.30, I63.311, I63.312, I63.319, I63.321, I63.322, I63.329, I63.331, I63.332, I63.339, I63.341, I63.342, I63.349, I63.39, I63.6, I66.01, I66.02, I66.03, I66.09, I66.11, I66.12, I66.13, I66.19, I66.21, I66.22, I66.23, I66.29, I66.3, I66.9, I66.40, I66.411, I66.412, I66.419, I66.421, I66.422, I66.429, I66.431, I66.432, I66.439, I66.441, I66.442, I66.449, I66.49, I66.01, I66.02, I66.03, I66.09, I66.11, I66.12, I66.13, I66.19, I66.21, I66.22, I66.23, I66.29, I66.3, I66.8, I66.9, I63.50, I63.511, I63.512, I63.519, I63.521, I63.522, I63.529, I63.531, I63.532, I63.539, I63.541, I63.542, I63.549, I63.59, I63.8, I63.9 |  |
|  |  | 435 | G45.0,G45.8,G45.1,G45.2,G46.0, G46.1, G46.2, G45.9, I67.841, I67.848 |  |
|  |  | 436 | I67.89 |  |
|  |  | 4371 | I67.81, I67.82, I67.89 |  |
|  |  | 4379 | I67.9 |  |

CABG, Coronary artery bypass graft; PCI, Percutaneous coronary intervention

Supplemental Table 13: The definition of important covariates and multivariate COX regression model

| Covariates | Categories | | | |
| --- | --- | --- | --- | --- |
| Sex (Questionnaire) | Women | | Men | |
| Age (Questionnaire) | 20~39 (y/o) | 40~59 (y/o) | | ≧60 (y/o) |
| Marital status (Questionnaire) | Married; Living with spouse | | Single/divorced/separated | |
| Education level (Questionnaire) | > 9 years | | ≦ 9 years | |
| Monthly income (Questionnaire) | ≥ 40000 NTD | | < 4000 NTD | |
| Parental history of CVD (Questionnaire) | Yes | | No | |
| Menopause status (Questionnaire) | Yes | | No | |
| Baseline hypertension  (NHIRD, ICD-9 and drug) | ICD-9*2 times or anti-HTN drug≧28 days or mean BP ≥ 140/80 mmHg | | No record of ICD-9*2 times and anti-HTN drug≧28 days and mean BP < 140/80 mmHg | |
| Baseline diabetes  (NHIRD, ICD-9 and drug) | ICD-9*2 times or anti-diabetes drug≧28 days or Fasting glucose ≥ 126mg/dL  or HbA1C ≥ 6.5 | | No record of ICD-9*2 times and anti-diabetes drug≧28 days and Fasting glucose < 126mg/dL or HbA1C < 6.5 | |
| Baseline hyperlipidemia  (NHIRD, ICD-9 and drug) | ICD-9*2 times or lipid lowering agent≧28  or TC ≥ 240 mg/dL days | | No record of ICD-9*2 times and lipid lowering agent≧28 days and TC < 240 mg/dL days | |
| History of hormone use (Questionnaire) | Yes | | No | |
| Abdominal obesity | waist circumflex ≥ 80 cm in women  and ≥ 90 cm in men | | waist circumflex < 80 cm in women  and < 90 cm in men | |

Model 1: adjusted for age and sex

Model 2: model 1, additionally education, average month income, marital status, parental history of CVD, menopause status and estrogen exposure

Model 3: model 2, additionally baseline HTN, baseline DM, history of hyperlipidemia, sBP, dBP, triglyceride, non-HDL, fasting glucose, HbA1c; The population attributable risk is the percentage of new cases of heart failure in the population attributable to nonadherence to the low-risk lifestyle factor

NHIRD: National health insurance research database; HTN: Hypertension; HbA1c: Hemoglobin A1c; TC: total cholesterol

Supplemental Table 14: Basic characteristics of the study participants at baseline, specified by adherence numbers of healthy lifestyle scores; (A). The weighted Mediterranean diet related healthy lifestyle score (0~17 points) (B) The WCRF/AICR recommended healthy lifestyle score (0~7 points) (C) Life’s Simple 7 score (0~14 points)

(A) The weighted Mediterranean diet related healthy lifestyle score (0~17 points)

Abbreviation: SD, standard deviation; BP, blood pressure; HDL, high density lipoprotein, LDL, low density lipoprotein; ANOVA and the chi-square tests were used to compare the means and proportions among groups

(B) The WCRF/AICR recommended healthy lifestyle score (0~7 points)

Abbreviation: SD, standard deviation; BP, blood pressure; HDL, high density lipoprotein, LDL, low density lipoprotein; ANOVA and the chi-square tests were used to compare the means and proportions among groups

(C) Life’s Simple 7 score (0~14 points)

Abbreviation: SD, standard deviation; BP, blood pressure; HDL, high density lipoprotein, LDL, low density lipoprotein; ANOVA and the chi-square tests were used to compare the means and proportions among group

Supplemental Table 15: The Spearman’s correlation coefficients between the four healthy lifestyle scores; (A) crude model (B) model with adjusted age and sex; MHL: Mediterranean diet related healthy lifestyle score; WCRF/AICR: World Cancer Research Fund/American Institute for Cancer Research

(A)

|  |  |  |  |  |
| --- | --- | --- | --- | --- |
|  | Simple MHL | Weighted MHL | WCRF/AICR | Life's Simple 7 |
| Simple MHL | 1 | 0.84 | 0.16 | 0.42 |
| *p* value |  | <0.001 | <0.001 | <0.001 |
| Weighted MHL | 0.84 | 1 | 0.04 | 0.21 |
| *p* value | <0.001 |  | <0.001 | <0.001 |
| WCRF/AICR | 0.16 | 0.04 | 1 | 0.31 |
| *p* value | <0.001 | <0.001 |  | <0.001 |
| Life's Simple 7 | 0.42 | 0.21 | 0.31 | 1 |
| *p* value | <0.001 | <0.001 | <0.001 |  |

(B)

|  |  |  |  |  |
| --- | --- | --- | --- | --- |
|  | Simple MHL | Weighted MHL | WCRF/AICR | Life's Simple 7 |
| Simple MHL | 1 | 0.86 | 0.16 | 0.37 |
| *p* value |  | <0.001 | <0.001 | <0.001 |
| Weighted MHL | 0.86 | 1 | 0.07 | 0.20 |
| *p* value | <0.001 |  | <0.001 | <0.001 |
| WCRF/AICR | 0.16 | 0.07 | 1 | 0.31 |
| *p* value | <0.001 | <0.001 |  | <0.001 |
| Life's Simple 7 | 0.37 | 0.20 | 0.31 | 1 |
| *p* value | <0.001 | <0.001 | <0.001 |  |

Supplemental Table 16 : Interrater agreement of different criteria of healthy lifestyle score by κ values (A), each cell presenting the κ when deleting this criterion (B); MHL: Mediterranean diet related healthy lifestyle score; WCRF/AICR: World Cancer Research Fund/American Institute for Cancer Research

(A)

|  |  |  |  |
| --- | --- | --- | --- |
| Kappa | Weighted MHL | WCRF/AICR | Life's Simple 7 |
| Simple MHL | 0.6 | -0.047 | 0.07 |
| *p* value | < 0.001 | 1 | < 0.001 |
| Weighted MHL |  | -0.05 | 0.06 |
| *p* value |  | 1 | < 0.001 |
| WCRF/AICR |  |  | 0.07 |
| *p* value |  |  | < 0.001 |

| (B) |  |  |
| --- | --- | --- |
| Healthy lifestyle scores | Kappa | P value |
| All 4 healthy lifestyle scores | 0.1 | < 0.001 |
| 3 healthy lifestyle scores without simple MHL | 0.15 | < 0.001 |
| 3 healthy lifestyle scores without weighted MHL | 0.21 | < 0.001 |
| 3 healthy lifestyle scores without WCRF/AICR | 0.01 | 0.012 |
| 3 healthy lifestyle scores without Life's Simple 7 | -0.006 | 0.91 |

Supplemental Table 17: The incidence cases, follow-up person-years, and the rates of cardiovascular disease events according to lifestyle factors and the hazard ratios and 95% confidence intervals

(A) According to the body mass index in 6048 for TWsHHH study participants (B) According to the Mediterranean diet score in 6048 for TWsHHH study participants (C) According to the time of physical activity in 6048 for TWsHHH study participants (D) According to the smoking status in 6048 for TWsHHH study participants (E) According to the frequency of alcohol consumption in 6048 for TWsHHH study participants;

(Model 1: adjusted for age and sex; Model 2: Model 1 + education, average month income, marital status, parental history of CVD, menopause status and estrogen exposure; Model 3: Model 2 + baseline HTN, baseline DM, history of hyperlipidemia, sBP, dBP, triglyceride, non-HDL, fasting glucose, HbA1c; The population attributable risk is the percentage of new cases of heart failure in the population attributable to nonadherence to the low-risk lifestyle factor.)

| BMI | <18.5 | 18.5~24.9 | |  | 25~29.9 |  |  | 30~34.9 |  |  | 35~39.9 |  |  | ≧ 40 |  |  | *P* of Logrank |  | | |
| --- | --- | --- | --- | --- | --- | --- | --- | --- | --- | --- | --- | --- | --- | --- | --- | --- | --- | --- | --- | --- |
| Cases | 10 | 243 |  |  | 165 |  |  | 25 |  |  | 5 |  |  | 1 |  |  |  |  |  |  |
| Pearson-year | 4639 | 47799 | |  | 16740.8 | |  | 2876 |  |  | 403 |  |  | 38.6 |  |  |  |  |  |  |
| Rates/1000 py | 2.2 | 5.1 |  |  | 9.9 |  |  | 8.7 |  |  | 12.4 |  |  | 25.9 |  |  | <.001 |  |  |  |
|  | HR | HR | 95% CI | | HR | 95% CI | | HR | 95% CI | | HR | 95% CI | | HR | 95% CI | | *P* of Trend Test | PAF | 95% CI | |
| Univariate | 1.00 | 2.31 | 1.19 | 4.51 | 4.62 | 2.36 | 9.06 | 3.62 | 1.65 | 7.95 | 4.93 | 1.52 | 16.01 | 40.46 | 8.74 | 187.36 | <.001 |  |  |  |
| Model 1 | 1.00 | 1.24 | 0.64 | 2.42 | 1.87 | 0.95 | 3.67 | 2.05 | 0.93 | 4.49 | 4.09 | 1.26 | 13.30 | 36.56 | 7.82 | 171.00 | <.001 |  |  |  |
| Model 2 | 1.00 | 1.23 | 0.63 | 2.40 | 1.79 | 0.91 | 3.51 | 1.87 | 0.85 | 4.11 | 3.36 | 1.02 | 11.10 | 40.22 | 8.53 | 189.66 | <.001 |  |  |  |
| Model 3 | 1.00 | 0.79 | 0.40 | 1.55 | 1.03 | 0.52 | 2.05 | 0.93 | 0.41 | 2.12 | 0.96 | 0.20 | 4.59 | 31.17 | 6.54 | 148.58 | 0.012 | 25.7 | 5.6 | 41.6 |

|  | 0~3 | 4~5 |  |  | 6~7 |  |  | 8~11 |  |  | *P* of Logrank |  | | |
| --- | --- | --- | --- | --- | --- | --- | --- | --- | --- | --- | --- | --- | --- | --- |
| Cases | 145 | 209 |  |  | 133 |  |  | 33 |  |  |  |  |  |  |
| Pearson-year | 13786 | 24958 |  |  | 23647 |  |  | 13246 |  |  |  |  |  |  |
| Rates/1000 py | 10.52 | 8.37 |  |  | 5.62 |  |  | 2.49 |  |  | <0.001 |  |  |  |
|  | HR | HR | 95% CI | | HR | 95% CI | | HR | 95% CI | | *P* of Trend Test | PAF | 95% CI | |
| Univariate | 1.00 | 0.79 | 0.62 | 1.01 | 0.51 | 0.39 | 0.67 | 0.20 | 0.12 | 0.31 | <0.001 |  |  |  |
| Model 1 | 1.00 | 0.88 | 0.69 | 1.13 | 0.73 | 0.56 | 0.96 | 0.40 | 0.25 | 0.63 | <0.001 |  |  |  |
| Model 2 | 1.00 | 0.90 | 0.71 | 1.15 | 0.78 | 0.59 | 1.03 | 0.42 | 0.27 | 0.67 | 0.002 |  |  |  |
| Model 3 | 1.00 | 0.92 | 0.71 | 1.21 | 0.83 | 0.61 | 1.12 | 0.35 | 0.20 | 0.61 | 0.008 | 23.7 | 6.7 | 37.6 |

| Physical activity Mins/week | 0 | 1~49 |  |  | 50~99 |  |  | 100~149 |  |  | >= 150 |  |  | *P* of Logrank |  | | |
| --- | --- | --- | --- | --- | --- | --- | --- | --- | --- | --- | --- | --- | --- | --- | --- | --- | --- |
| Cases | 234 | 17 |  |  | 24 |  |  | 30 |  |  | 215 |  |  |  |  |  |  |
| Pearson-year | 36254 | 5690 |  |  | 7170 |  |  | 5130 |  |  | 21393.1 | |  |  |  |  |  |
| Rates/1000 py | 6.45 | 3.0 |  |  | 3.35 |  |  | 5.85 |  |  | 10.1 |  |  | <.001 |  |  |  |
|  | HR | HR | 95% CI | | HR | 95% CI | | HR | 95% CI | | HR | 95% CI | | *P* of Trend Test | PAF | 95% CI | |
| Univariate | 1 | 0.45 | 0.26 | 0.77 | 0.55 | 0.36 | 0.86 | 0.82 | 0.53 | 1.27 | 1.45 | 1.17 | 1.8 | <.001 |  |  |  |
| Model 1 | 1 | 0.60 | 0.35 | 1.04 | 0.75 | 0.48 | 1.17 | 0.86 | 0.56 | 1.32 | 1.04 | 0.84 | 1.3 | 0.70 |  |  |  |
| Model 2 | 1 | 0.65 | 0.38 | 1.13 | 0.80 | 0.51 | 1.25 | 0.94 | 0.61 | 1.45 | 1.12 | 0.9 | 1.4 | 0.26 |  |  |  |
| Model 3 | 1 | 0.60 | 0.33 | 1.09 | 0.82 | 0.50 | 1.34 | 0.91 | 0.57 | 1.45 | 1.08 | 0.84 | 1.38 | 0.33 | -5.3 | -18 | 6 |

| Divided groups | 0 | 1^st^ group |  |  | 2^nd^ group |  |  | 3^rd^ group |  |  | 4^th^ group |  |  | *P* of Logrank |  | | |
| --- | --- | --- | --- | --- | --- | --- | --- | --- | --- | --- | --- | --- | --- | --- | --- | --- | --- |
| Cases | 120 | 19 |  |  | 2 |  |  | 43 |  |  | 336 |  |  |  |  |  |  |
| Pearson-year | 8998 | 9299.4 | |  | 260.7 | |  | 2397 | |  | 54600.5 | |  |  |  |  |  |
| Rates/1000 py | 13.34 | 2.0 |  |  | 7.67 |  |  | 17.9 |  |  | 6.15 |  |  | <.001 |  |  |  |
|  | HR | HR | 95% CI | | HR | 95% CI | | HR | 95% CI | | HR | 95% CI | | *P* of Trend Test | PAF | 95% CI | |
| Univariate | 1 | 0.15 | 0.09 | 0.26 | 0.79 | 0.20 | 3.20 | 1.19 | 0.79 | 1.81 | 0.43 | 0.34 | 0.54 | <.001 |  |  |  |
| Model 1 | 1 | 1.01 | 0.58 | 1.76 | 1.54 | 0.38 | 6.24 | 1.01 | 0.66 | 1.53 | 0.97 | 0.74 | 1.27 | 0.57 |  |  |  |
| Model 2 | 1 | 0.98 | 0.56 | 1.72 | 1.87 | 0.46 | 7.64 | 1.10 | 0.72 | 1.67 | 1.03 | 0.79 | 1.35 | 0.98 |  |  |  |
| Model 3 | 1 | 1.03 | 0.56 | 1.93 | 2.51 | 0.61 | 10.33 | 0.97 | 0.61 | 1.56 | 0.90 | 0.66 | 1.21 | 0.54 | 2.4 | -4.7 | 8.9 |

Note: 0 group: current smoking >=20 years; 1st group: current smoking < 20 years; 2ne group: quit smoking < 1 year; 3rd group: quit smoking >= 1 year; 4th group: never or smoking less than 100 cigarettes

|  | Never | Few |  |  | Frequency |  |  | *P* of Logrank |  | | |
| --- | --- | --- | --- | --- | --- | --- | --- | --- | --- | --- | --- |
| Cases | 403 | 92 |  |  | 25 |  |  |  |  |  |  |
| Pearson-year | 54756 | 17146.2 | |  | 3734 |  |  |  |  |  |  |
| Rates/1000 py | 7.36 | 5.37 |  |  | 6.69 |  |  | 0.022 |  |  |  |
|  | HR | HR | 95% CI | | HR | 95% CI | | *P* of Trend Test | PAF | 95% CI | |
| Univariate | 1 | 0.85 | 0.66 | 1.09 | 1 | 0.63 | 1.57 | 0.035 |  |  |  |
| Model 1 | 1 | 0.77 | 0.59 | 0.99 | 0.59 | 0.38 | 0.94 | <.001 |  |  |  |
| Model 2 | 1 | 0.76 | 0.59 | 0.98 | 0.59 | 0.37 | 0.93 | <.001 |  |  |  |
| Model 3 | 1 | 0.79 | 0.6 | 1.05 | 0.5 | 0.29 | 0.87 | 0.001 | 41.1 | 18.1 | 57.6 |

Supplemental Table 18: The incidence cases, follow-up person-years, and the rates of cardiovascular disease events according to lifestyle factors and the hazard ratios and 95% confidence intervals; (A) The weighted MHL healthy lifestyle score, according to the score quartiles (B) The WCRF/AICR recommended healthy lifestyle score, according to the numbers of the score (C) Life’s Simple 7, according to the numbers of score (0~14 points); MHL: Mediterranean diet related healthy lifestyle score; WCRF/AICR: World Cancer Research Fund/American Institute for Cancer Research

(Note: Model 1: adjusted for age and sex; Model 2: Model 1, additionally education, average month income, marital status, parental history of CVD, menopause status and estrogen exposure; Model 3: Model 2 + baseline HTN, baseline DM, history of hyperlipidemia, sBP, dBP, triglyceride, non-HDL, fasting glucose, HbA1c; The population attributable risk is the percentage of new cases of heart failure in the population attributable to nonadherence to the low-risk lifestyle factor.)

(A)

|  | 0~4 | 5 |  |  | 6~7 |  |  | 8~17 |  |  | *P* of Logrank |  | | |
| --- | --- | --- | --- | --- | --- | --- | --- | --- | --- | --- | --- | --- | --- | --- |
| Cases | 312 | 89 |  |  | 82 |  |  | 37 |  |  |  |  |  |  |
| Pearson-year | 31049 | 12213.6 |  |  | 20456.2 |  |  | 11836.4 |  |  |  |  |  |  |
| Rates/1000 py | 10.05 | 7.29 |  |  | 4.01 |  |  | 3.13 |  |  | <0.001 |  |  |  |
|  | HR | HR | 95% CI | | HR | 95% CI | | HR | 95% CI | | *P* of Trend Test | PAF | 95% CI | |
| Univariate | 1.00 | 0.86 | 0.64 | 1.15 | 0.72 | 0.53 | 0.98 | 0.45 | 0.29 | 0.70 | <0.001 |  |  |  |
| Model 1 | 1.00 | 0.80 | 0.61 | 1.05 | 0.62 | 0.46 | 0.82 | 0.47 | 0.32 | 0.69 | <0.001 |  |  |  |
| Model 2 | 1.00 | 0.82 | 0.63 | 1.08 | 0.67 | 0.51 | 0.90 | 0.49 | 0.34 | 0.73 | <0.001 |  |  |  |
| Model 3 | 1.00 | 0.82 | 0.61 | 1.11 | 0.69 | 0.51 | 0.95 | 0.44 | 0.28 | 0.68 | <0.001 | 34.3 | 17.8 | 47.4 |

(B)

|  | <3 | 3~ <4 |  |  | 4~ <5 |  |  | 5~7 |  |  | *P* of Logrank |  | | |
| --- | --- | --- | --- | --- | --- | --- | --- | --- | --- | --- | --- | --- | --- | --- |
| Cases | 55 | 94 |  |  | 193 |  |  | 178 |  |  |  |  |  |  |
| Pearson-year | 14391 | 19563.7 |  |  | 24508.2 |  |  | 17174.1 |  |  |  |  |  |  |
| Rates/1000 py | 3.82 | 4.8 |  |  | 7.87 |  |  | 10.36 |  |  | <0.001 |  |  |  |
|  | HR | HR | 95% CI | | HR | 95% CI | | HR | 95% CI | | *P* of Trend Test | PAF | 95% CI | |
| Univariate | 1 | 1.21 | 0.84 | 1.75 | 1.97 | 1.41 | 2.74 | 2.52 | 1.8 | 3.53 | <.001 |  |  |  |
| Model 1 | 1 | 0.99 | 0.69 | 1.44 | 0.88 | 0.63 | 1.23 | 0.89 | 0.63 | 1.26 | 0.38 |  |  |  |
| Model 2 | 1 | 0.94 | 0.65 | 1.36 | 0.83 | 0.59 | 1.17 | 0.88 | 0.62 | 1.24 | 0.49 |  |  |  |
| Model 3 | 1 | 1.33 | 0.75 | 2.34 | 1.32 | 0.76 | 2.29 | 1.44 | 0.82 | 2.51 | 0.30 | -6.2 | -19 | 4.9 |

(C)

|  | 0~6 | 7~9 |  |  | 10~12 |  |  | 13~14 |  |  | *P* of Logrank |  | | |
| --- | --- | --- | --- | --- | --- | --- | --- | --- | --- | --- | --- | --- | --- | --- |
| Cases | 36 | 194 |  |  | 255 |  |  | 35 |  |  |  |  |  |  |
| Pearson-year | 1801.91 | 16826.4 |  |  | 42851.6 |  |  | 14157 |  |  |  |  |  |  |
| Rates/1000 py | 20.0 | 11.5 |  |  | 6.0 |  |  | 2.47 |  |  | <0.001 |  |  |  |
|  | HR | HR | 95% CI | | HR | 95% CI | | HR | 95% CI | | *P* of Trend Test | PAF | 95% CI | |
| Univariate | 1 | 0.63 | 0.40 | 0.99 | 0.36 | 0.23 | 0.56 | 0.16 | 0.09 | 0.27 | <0.001 |  |  |  |
| Model 1 | 1 | 0.54 | 0.35 | 0.85 | 0.37 | 0.24 | 0.58 | 0.21 | 0.12 | 0.37 | <0.001 |  |  |  |
| Model 2 | 1 | 0.60 | 0.38 | 0.94 | 0.43 | 0.27 | 0.66 | 0.27 | 0.15 | 0.47 | <0.001 |  |  |  |
| Model 3 | 1 | 0.84 | 0.51 | 1.39 | 0.86 | 0.50 | 1.48 | 0.60 | 0.29 | 1.24 | 0.02 | 24.5 | 3.1 | 41.2 |

Supplemental Table 19: Sensitivity test of the incidence cases, follow-up person-years, and the rates of cardiovascular disease events and the hazard ratios and 95% confidence intervals

(A) The simple Mediterranean diet related healthy lifestyle score without alcohol intake (B) The weighted Mediterranean diet related healthy lifestyle score without alcohol intake (C) The simple Mediterranean diet related healthy lifestyle score, substitute of body mass index by waist circumflex; (D)The weighted Mediterranean diet related healthy lifestyle score, substitute of body mass index by waist circumflex (E) The weighted each lifestyle as categorical factor rather than dichotomous one to calculated the weighted Mediterranean diet related healthy lifestyle score

(A) The simple Mediterranean diet related healthy lifestyle score without alcohol intake

|  |  |  |  |  |  |  |  |  |  |  |  |
| --- | --- | --- | --- | --- | --- | --- | --- | --- | --- | --- | --- |
| Lifestyle score | 0~1 | 2 |  |  | 3 |  |  | 4 |  |  | *P* of Logrank |
| N | 1472 | 2395 |  |  | 1750 |  |  | 425 |  |  |  |
| Cases | 207 | 218 |  |  | 86 |  |  | 9 |  |  |  |
| Pearson-year | 17450 | 29666 |  |  | 22721 |  |  | 5717.3 |  |  |  |
| Rates/1000 py | 11.86 | 7.35 |  |  | 3.79 |  |  | 1.57 |  |  | <0.001 |
|  | HR | HR | 95% CI | | HR | 95% CI | | HR | 95% CI | | Trend Test |
| Univariate | 1 | 0.55 | 0.44 | 0.68 | 0.28 | 0.21 | 0.37 | 0.12 | 0.06 | 0.25 | <0.001 |
| Model 1 | 1 | 0.7 | 0.56 | 0.87 | 0.52 | 0.39 | 0.7 | 0.35 | 0.16 | 0.76 | <0.001 |
| Model 2 | 1 | 0.73 | 0.59 | 0.92 | 0.57 | 0.43 | 0.77 | 0.41 | 0.19 | 0.88 | <0.001 |
| Model 3 | 1 | 0.76 | 0.6 | 0.98 | 0.59 | 0.42 | 0.82 | 0.47 | 0.2 | 1.07 | 0.003 |

(B) The weighted Mediterranean diet related healthy lifestyle score without alcohol intake

|  |  |  |  |  |  |  |  |  |  |  |  |
| --- | --- | --- | --- | --- | --- | --- | --- | --- | --- | --- | --- |
|  | Q1 | Q2 |  |  | Q3 |  |  | Q4 |  |  | *P* of Logrank |
| N | 2772 | 1038 |  |  | 1570 |  |  | 662 |  |  |  |
| Cases | 332 | 91 |  |  | 80 |  |  | 17 |  |  |  |
| Pearson-year | 33354 | 13019 |  |  | 20392 |  |  | 8790 |  |  |  |
| Rates/1000 py | 9.95 | 6.99 |  |  | 3.92 |  |  | 1.93 |  |  | <0.001 |
|  | HR | HR | 95% CI | | HR | 95% CI | | HR | 95% CI | | Trend Test |
| Univariate | 1 | 0.69 | 0.53 | 0.9 | 0.36 | 0.27 | 0.47 | 0.2 | 0.12 | 0.34 | <0.001 |
| Model 1 | 1 | 0.76 | 0.58 | 1 | 0.61 | 0.46 | 0.82 | 0.43 | 0.25 | 0.73 | <0.001 |
| Model 2 | 1 | 0.79 | 0.6 | 1.03 | 0.67 | 0.5 | 0.9 | 0.46 | 0.27 | 0.78 | <0.001 |
| Model 3 | 1 | 0.79 | 0.59 | 1.06 | 0.71 | 0.52 | 0.97 | 0.41 | 0.23 | 0.77 | 0.003 |

(C) The simple Mediterranean diet related healthy lifestyle score, substitute of body mass index by waist circumflex

|  | O~1 | 2 |  |  | 3 |  |  | 4~5 |  |  | *P* of Logrank |  | | |
| --- | --- | --- | --- | --- | --- | --- | --- | --- | --- | --- | --- | --- | --- | --- |
| Cases | 249 | 205 |  |  | 56 |  |  | 10 |  |  |  |  |  |  |
| Pearson-year | 19303 | 28771.5 | |  | 21790.6 | |  | 5689.7 | |  |  |  |  |  |
| Rates/1000 py | 12.9 | 7.1 |  |  | 2.57 |  |  | 1.76 |  |  | < 0.001 |  |  |  |
|  | HR | HR | 95% CI | | HR | 95% CI | | HR | 95% CI | | *P* of Trend Test | PAF | 95% CI | |
| Univariate | 1.00 | 0.55 | 0.45 | 0.68 | 0.21 | 0.16 | 0.29 | 0.13 | 0.07 | 0.27 | < 0.001 |  |  |  |
| Model 1 | 1.00 | 0.78 | 0.63 | 0.97 | 0.44 | 0.32 | 0.60 | 0.36 | 0.18 | 0.73 | < 0.001 |  |  |  |
| Model 2 | 1.00 | 0.81 | 0.65 | 1.00 | 0.48 | 0.35 | 0.67 | 0.41 | 0.20 | 0.83 | < 0.001 |  |  |  |
| Model 3 | 1.00 | 0.86 | 0.68 | 1.10 | 0.54 | 0.38 | 0.77 | 0.45 | 0.21 | 0.98 | < 0.001 |  |  |  |

(D) The weighted Mediterranean diet related healthy lifestyle score, substitute of body mass index by waist circumflex

|  | 0~4 | 5 |  |  | 6~7 |  |  | 8~17 |  |  | *P* of Logrank |  | | |
| --- | --- | --- | --- | --- | --- | --- | --- | --- | --- | --- | --- | --- | --- | --- |
| Cases | 241 | 194 |  |  | 52 |  |  | 33 |  |  |  |  |  |  |
| Pearson-year | 18822.4 | 26477.9 | |  | 18515.8 | |  | 11739 | |  |  |  |  |  |
| Rates/1000 py | 12.8 | 7.33 |  |  | 2.81 |  |  | 2.81 |  |  | < 0.001 |  |  |  |
|  | HR | HR | 95% CI | | HR | 95% CI | | HR | 95% CI | | *P* of Trend Test | PAF | 95% CI | |
| Univariate | 1.00 | 0.57 | 0.46 | 0.71 | 0.25 | 0.18 | 0.34 | 0.22 | 0.15 | 0.33 | < 0.001 |  |  |  |
| Model 1 | 1.00 | 0.81 | 0.65 | 1.01 | 0.51 | 0.37 | 0.71 | 0.41 | 0.27 | 0.62 | < 0.001 |  |  |  |
| Model 2 | 1.00 | 0.85 | 0.68 | 1.06 | 0.57 | 0.41 | 0.80 | 0.44 | 0.29 | 0.66 | < 0.001 |  |  |  |
| Model 3 | 1.00 | 0.92 | 0.72 | 1.18 | 0.67 | 0.47 | 0.95 | 0.40 | 0.25 | 0.65 | < 0.001 |  |  |  |

(E) The weighted each lifestyle as categorical factor rather than dichotomous one to calculated the weighted Mediterranean diet related healthy lifestyle score

|  |  |  |  |  |  |  |  |  |  |  |  |
| --- | --- | --- | --- | --- | --- | --- | --- | --- | --- | --- | --- |
|  | Q1 | Q2 |  |  | Q3 |  |  | Q4 |  |  | *P* of Logrank |
| N | 1740 | 1294 |  |  | 1328 |  |  | 1351 |  |  |  |
| Cases | 138 | 100 |  |  | 111 |  |  | 100 |  |  |  |
| Pearson-year | 21955 | 16265 |  |  | 16922 |  |  | 17273 |  |  |  |
| Rates/1000 py | 6.29 | 6.15 |  |  | 6.56 |  |  | 5.79 |  |  | 0.84 |
|  | HR | HR | 95% CI | | HR | 95% CI | | HR | 95% CI | | Trend Test |
| Univariate | 1 | 0.94 | 0.72 | 1.22 | 0.94 | 0.72 | 1.23 | 0.78 | 0.59 | 1.03 | 0.68 |
| Model 1 | 1 | 1.08 | 0.83 | 1.41 | 0.89 | 0.69 | 1.17 | 0.72 | 0.54 | 0.95 | 0.031 |
| Model 2 | 1 | 1.12 | 0.86 | 1.46 | 0.92 | 0.7 | 1.21 | 0.76 | 0.57 | 1.01 | 0.09 |
| Model 3 | 1 | 1.11 | 0.83 | 1.48 | 0.78 | 0.58 | 1.05 | 0.69 | 0.5 | 0.95 | 0.018 |

Supplemental Figure 1: Flow-chart of study design and participants exclusion criteria

Supplemental Figure 2: Kaplan-Meir survival curves for the risk of cardiovascular specified in the study participants among participants stratified by the numbers of healthy lifestyle factors (A) by simple Mediterranean diet related healthy lifestyle score (B) by Mediterranean diet related healthy lifestyle score (C) by WCRF/AICR recommended lifestyle score (D) by Life’s Simple 7; MHL: Mediterranean diet related healthy lifestyle score; WCRF/AICR: World Cancer Research Fund/American Institute for Cancer Research


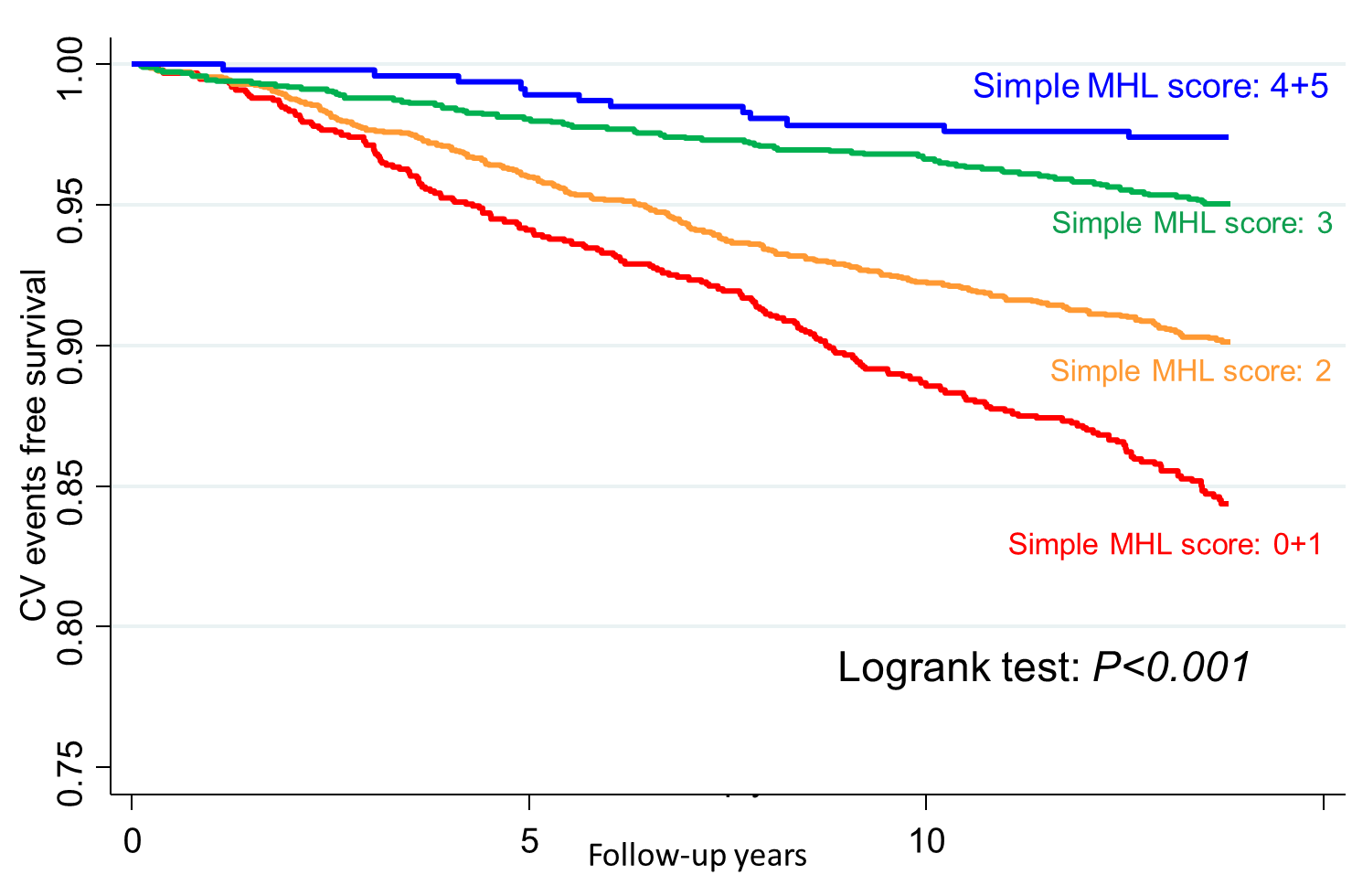


(B)


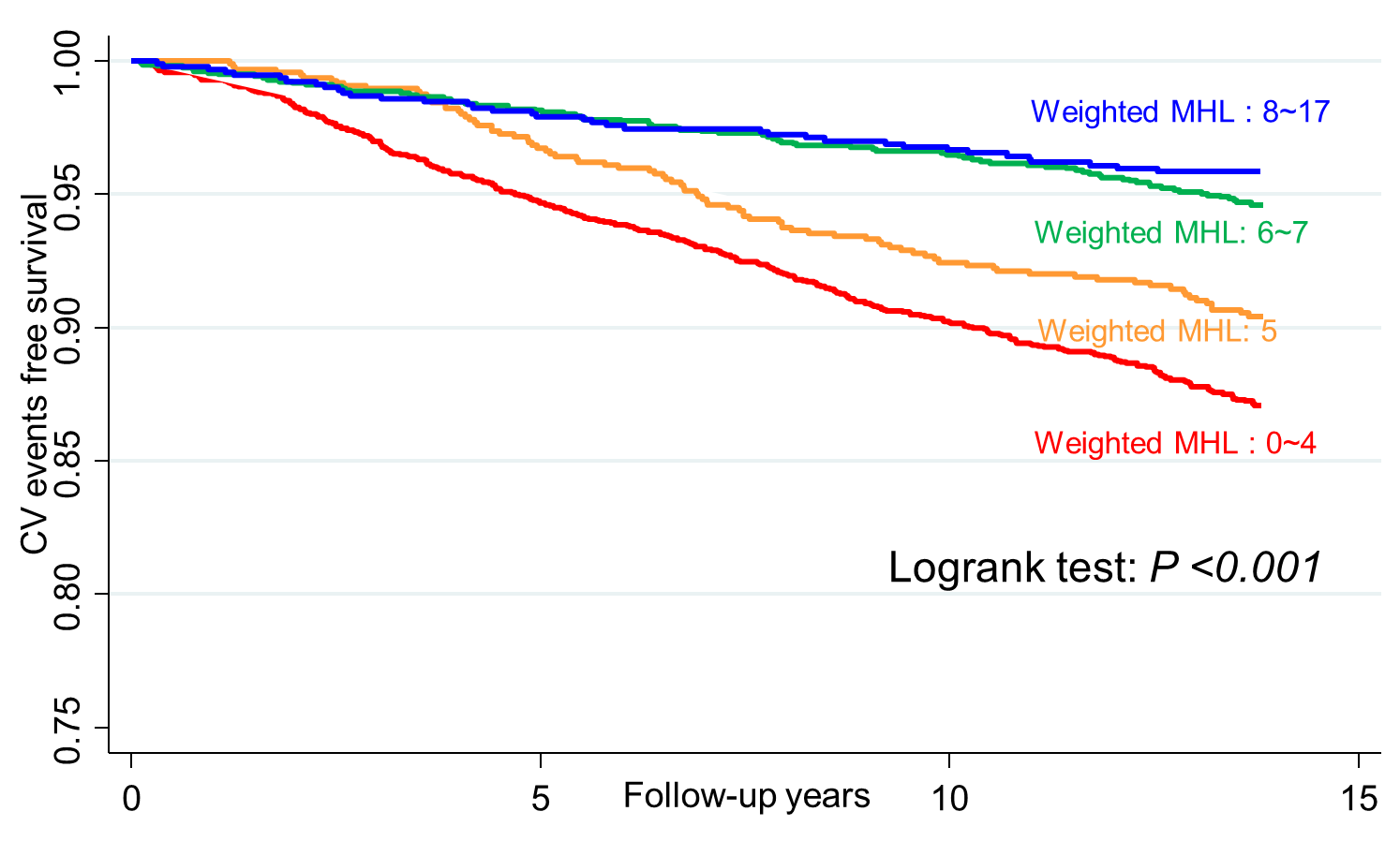


(C)

(D)

Supplemental Figure 3: The log(-log(survival time)) versus log of CVD event-free survival time including time independent covariates

1. CVD event-free survival time of simple Mediterranean diet related healthy lifestyle score (B) CVD event-free survival time of weighted Mediterranean diet related healthy lifestyle score (C) CVD event-free survival time of WCRF/AICR recommended lifestyle score (D) CVD event-free survival time of Life’s Simple 7 (E) CVD event-free survival time of body mass index (E) CVD event-free survival time of Mediterranean diet score (F) CVD event-free survival time of physical activity (G) CVD event-free survival time of Smoking status (H) CVD event-free survival time of alcohol consumption (I) CVD event-free survival time of ever, former or non-smoking status

(A)


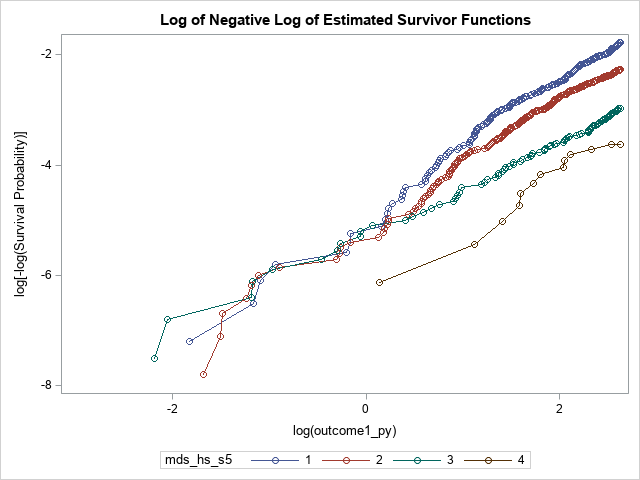


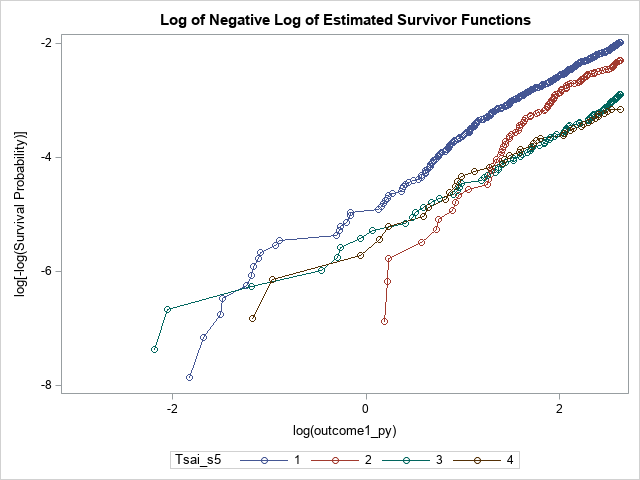


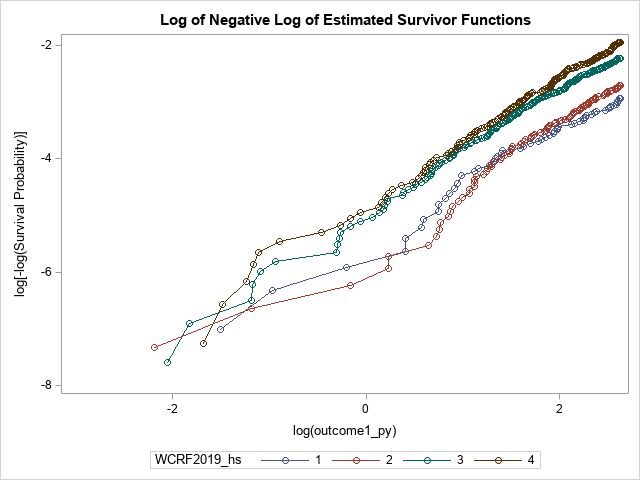


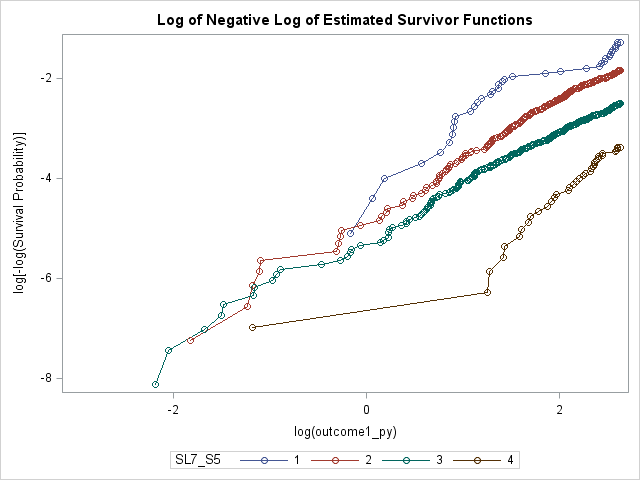


(E)


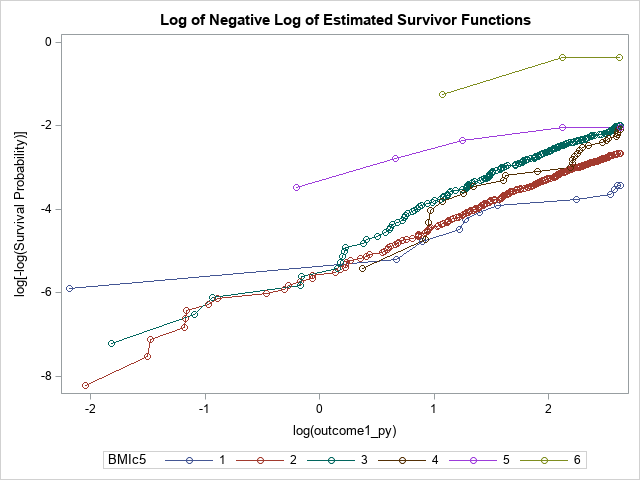


(F)


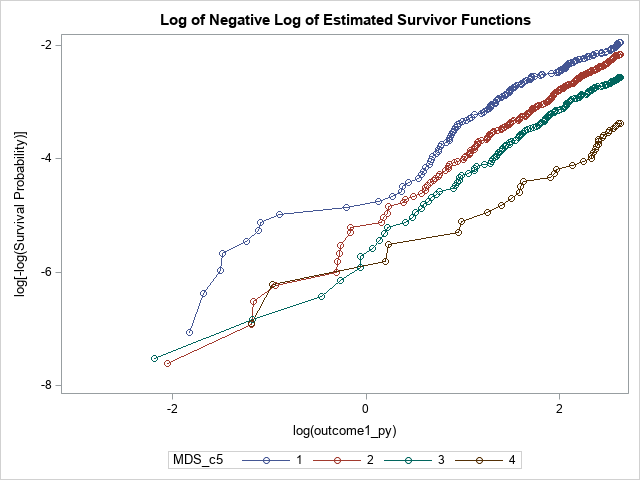


(G)


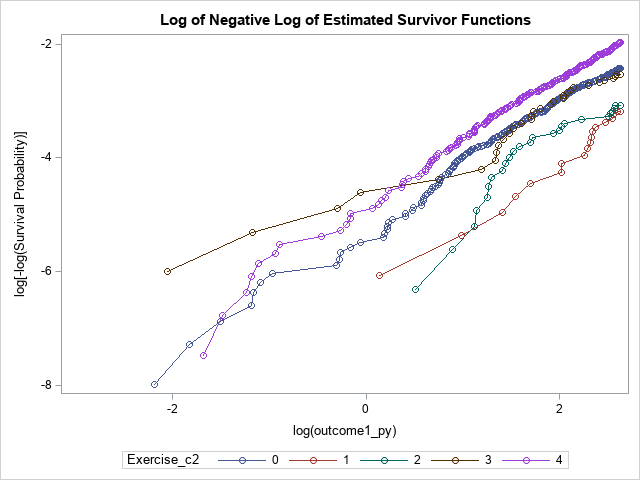


(H)


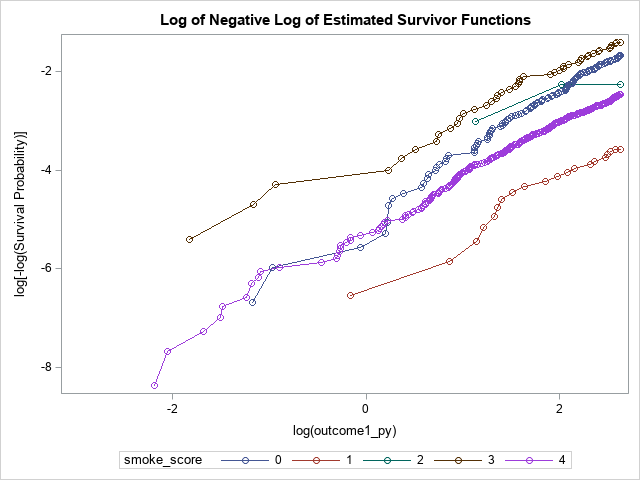


(I)


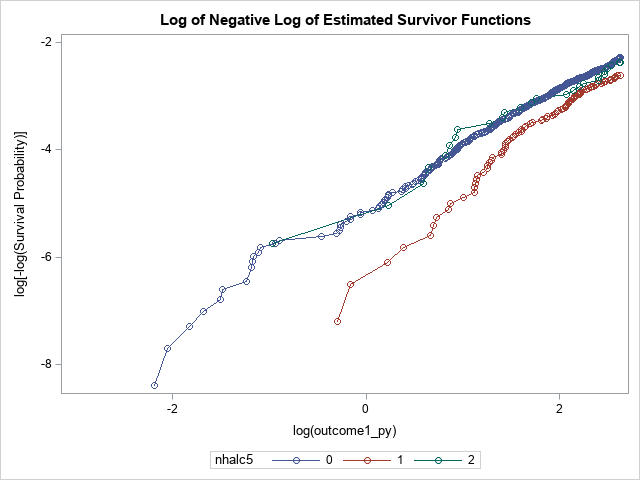


Supplemental Figure 4: Hazard ratios for cardiovascular disease with categories according to the numbers of healthy lifestyle factors among participants stratified by age < 60 y/o and ≥ 60 y/o

(A) Simple Mediterranean diet related healthy lifestyle score (B) Weighted Mediterranean diet related healthy lifestyle score (C) WCRF/AICR recommended healthy lifestyle score (D) Life’s Simple 7 score

(A)

*P for* interaction= 0.07


(C)

(D)

Supplemental Figure 5: Calibration plot of predicted mean 12.5.following-up years cardiovascular disease (CVD) risk within deciles against the observed 12.5 following-up years CVD risk in the TWsHHH data (N=6048). Data are plotted among participants stratified by lifestyle scores

(A) Simple Mediterranean diet related healthy lifestyle score (B) Weighted Mediterranean diet related healthy lifestyle score (C) WCRF/AICR recommended healthy lifestyle score (D) Life’s Simple 7 score

(A)


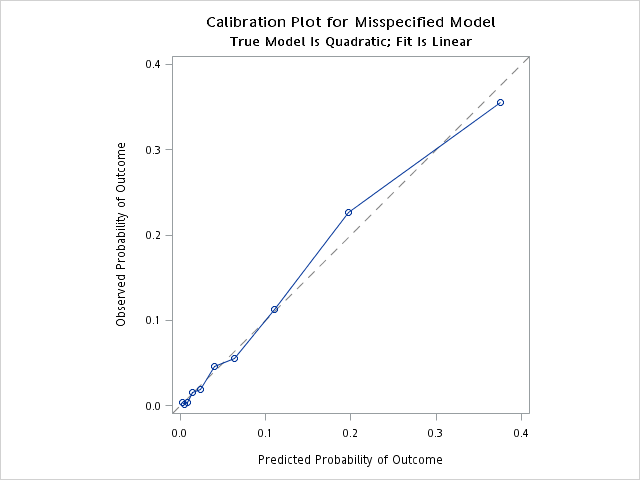


(B)


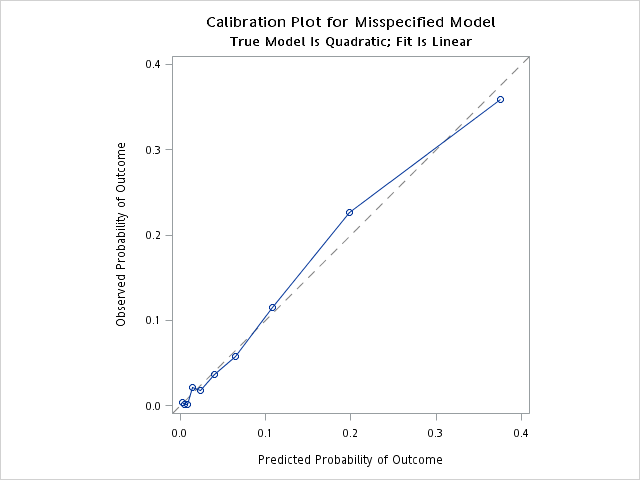


(C)


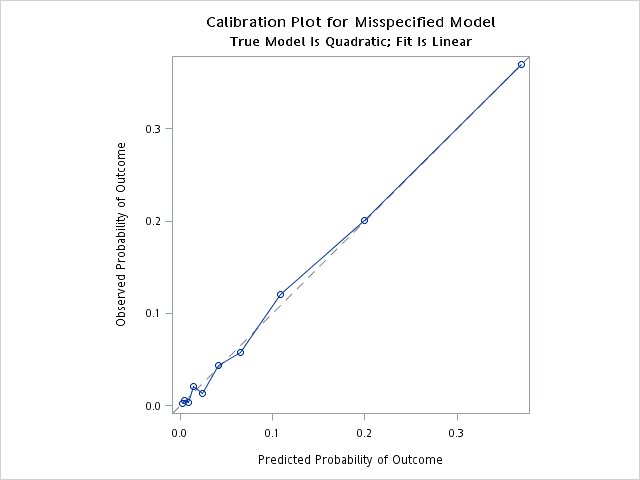


(D)


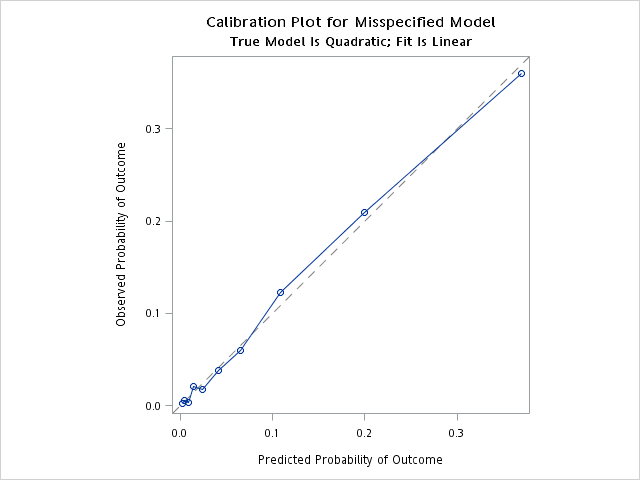


Supplemental Figure 6: Receiver-operating characteristic curves for various models applied to the study population; MHL: Mediterranean diet related healthy lifestyle score; WCRF/AICR: World Cancer Research Fund/American Institute for Cancer Research

0.00

0.25

0.50

0.75

1.00

Sensitivity

0.00

0.25

0.50

0.75

1.00

1-Specificity

Clasical AUC: 0.85

Weighted MHL score: 0.86

Simple MHL score: 0.86

WCRF/AICR score: 0.85

Life’s Simple 7: 0.85

Reference

*P_AUC_* = 0.048
